# Identifying pathways to increased volunteering in older US adults

**DOI:** 10.1038/s41598-022-16912-x

**Published:** 2022-07-27

**Authors:** Julia S. Nakamura, Matthew T. Lee, Frances S. Chen, Yeeun Archer Lee, Linda P. Fried, Tyler J. VanderWeele, Eric S. Kim

**Affiliations:** 1 Department of Psychology, University of British Columbia, 2136 West Mall, Vancouver, BC V6T 1Z4 Canada; 2 Institute for Studies of Religion, Baylor University, Waco, TX USA; 3 Human Flourishing Program, Institute for Quantitative Social Science, Harvard University, Cambridge, MA USA; 4 Mailman School of Public Health, Columbia University, New York City, NY USA; 5 Department of Biostatistics, Harvard T.H. Chan School of Public Health, Boston, MA USA; 6 Department of Epidemiology, Harvard T.H. Chan School of Public Health, Boston, MA USA; 7 Lee Kum Sheung Center for Health and Happiness, Harvard T.H. Chan School of Public Health, Boston, MA USA

**Keywords:** Epidemiology, Public health, Human behaviour

## Abstract

While growing evidence documents strong associations between volunteering and improved health and well-being outcomes, less is known about the health and well-being factors that lead to increased volunteering. Using data from 13,771 participants in the Health and Retirement Study (HRS)—a diverse, longitudinal, and national sample of older adults in the United States—we evaluated a large range of candidate predictors of volunteering. Specifically, using generalized linear regression models with a lagged exposure-wide approach, we evaluated if *changes* in 61 predictors spanning physical health, health behaviors, and psychosocial well-being (over a 4-year follow-up between t_0_; 2006/2008 and t_1_; 2010/2012) were associated with volunteer activity four years later (t_2_; 2014/2016). After adjusting for a rich set of covariates, certain changes in some health behaviors (e.g., physical activity ≥ 1x/week), physical health conditions (e.g., physical functioning limitations, cognitive impairment), and psychosocial factors (e.g., purpose in life, constraints, contact with friends, etc.) were associated with increased volunteering four years later. However, there was little evidence that other factors were associated with subsequent volunteering. Changes in several indicators of physical health, health behaviors, and psychosocial well-being may predict increased volunteering, and these factors may be novel targets for interventions and policies aiming to increase volunteering in older adults.

## Introduction

In light of decreasing volunteering rates across the nation^1^, and to enhance societal and individual well-being, researchers are seeking to understand and promote pathways that increase volunteering. There are four key reasons it is critical to study health and well-being factors that may predict subsequent volunteering. First, volunteers have a substantial impact on our economy. In 2018, approximately 22 million Baby Boomers volunteered 2.2 billion hours in the United States, generating $54.3 billion in value to their communities^2^. Second, growing evidence also suggests that volunteers themselves reap health and well-being benefits from their volunteering^3^. Third, the COVID-19 pandemic has brought to light substantial and persistent health disparities as the virus has not affected all sociodemographic groups equally. As inequality grows, there is a need to implement society-wide policies, health care programs, and prevention/intervention efforts that improve the lives of all members of society across the sociodemographic spectrum. While formal volunteering may not be accessible to all sociodemographic groups, for those who are willing and able to volunteer ~ 2 h per week^4,5^, volunteering offers a unique route to improved health and well-being. Fourth, populations are rapidly aging in many countries throughout the world^6^. In the United States, the population of older adults (aged ≥ 65 years) is projected to increase nearly 50% in the next 15 years^7^. With rapid population aging, researchers and policymakers are increasingly interested in health assets that enhance our society’s health and well-being^8,9^. Indeed, the health benefits of volunteering further benefit society through reduced health care costs, supplements to workforce shortages, and the promotion of health that facilitate independence and aging in place. Thus, physicians and policymakers are being encouraged to consider “prescribing” volunteering as it simultaneously benefits society and individuals^4,10,11^ to promote health across the age span, with consideration to local, community-based, and culturally relevant volunteering programs and initiatives^12–14^.

While a fairly large literature exists on how sociodemographic factors (e.g., age, education)^11,15^ predict subsequent volunteering (see *Discussion*), we focused on non-sociodemographic candidate predictors. Growing evidence documents how health behaviors (e.g. more physical activity)^16^, indicators of physical health (e.g., enhanced functional- and cognitive-functioning)^17,18^, psychological factors (e.g., more perceived behavioral control (i.e., perceived ease or difficulty of performing volunteering))^19–22^, and social factors (e.g., larger social networks, more social capital)^11,15,17^, have been positively associated with volunteering. However, in other studies, indicators of physical health (e.g., number of comorbidities)^23^ are not associated with volunteering. Further, some predictors are linked to conflicting findings (e.g., both increased depression^24^ and decreased depressive symptoms^25,26^ are associated with increased volunteering).

These existing studies have given us great insights into potential predictors of increased volunteering, but they remain limited from a causal inference point of view. First, while an increasing number of studies use longitudinal designs^17^, many prior studies are cross-sectional, making it difficult or impossible to address issues of potential reverse causation^27^. Second, most studies evaluate predictors of volunteering accumulated across the life-course, rather than changes in predictors. Thus, in these studies, we are unable to see the dynamic associations between volunteering and *changes* in key predictors. Third, many studies do not adequately adjust for key potential confounders. Fourth, most studies only evaluate a limited range of predictors. Fifth, many studies use data from small samples or very specific subpopulations, and results may not generalize to broader populations.

In our study, we asked a slightly different question than most past observational studies have asked: how might change in a predictor lead to change in volunteering behavior over time? To begin addressing this question, we used a new *lagged exposure-wide* analytic approach which is described further in the “Statistical analysis” section^28^. This allowed us to prospectively test if *changes* in 61 predictors spanning physical health, health behaviors, and psychosocial well-being factors (over a 4-year period) were associated with subsequent volunteering (4 years later). Exposure-wide analyses are a hypothesis-generating, data-driven approach aimed at discovering promising predictors of volunteering, which may then undergo further investigation in future studies. We chose these 61 predictors for four main reasons. First, because they are frequently included in the conceptualization of key gerontological models that characterize the antecedents, processes, and outcomes that foster people’s ability to age well^29–33^. Second, most of our candidate predictors have either shown evidence that they can be modified through intervention, or are likely modifiable with further research. Third was a very practical reason, that these predictors are available in the Health and Retirement Study (HRS) dataset. Many other factors (e.g., ease of transportation) may influence individuals’ ability or motivation to volunteer but are not available in the HRS dataset. Fourth, prior studies assessing predictors of volunteering have assessed micro-level contributors (incentive factors) and inhibitors (obstructive factors) of volunteering at the individual level in several key domains (health-related, psychological, etc.), creating a strong foundation for us to now assess how *changes* in these individual-level factors are associated with subsequent volunteering^34^. Future studies should further investigate other potential predictors (e.g., meso- and macro-level factors, factors available outside of HRS, etc.)^34^.

## Methods

### Study population

We used data from the Health and Retirement Study (HRS)—a national panel study of adults aged > 50 in the United States. In 2006, when psychosocial data were first collected, about half of HRS respondents completed an enhanced face-to-face (EFTF) interview (the other half of respondents were assessed in 2008). Participants then completed a psychosocial questionnaire (response rates: 88% in 2006, 84% in 2008)^35^ which they mailed to the University of Michigan upon completion. These sub-cohorts alternate reporting on psychosocial factors, with each participant reporting psychosocial data every 4 years. To increase sample size and statistical power, data from the 2006 and 2008 sub-cohorts were combined to create the pre-baseline wave. Participants were excluded if they did not report psychosocial data in this pre-baseline wave since over half of the study predictors were psychosocial factors. This resulted in a final sample of 13,771 participants.

We used data from three time points spaced four years apart. Covariates were assessed in the pre-baseline wave (t_0_; 2006/2008), candidate predictors were assessed in the baseline wave (t_1_; 2010/2012), and our outcome (volunteering) was assessed in the outcome wave (t_2_; 2014/2016). The HRS (see further study documentation on the HRS website: http://hrsonline.isr.umich.edu/) is sponsored by the National Institute on Aging (NIA U01AG009740) and conducted by the University of Michigan^36^. The HRS has been approved by various ethics committees, including the University of Michigan Institutional Review Board. Informed consent has been obtained from all participants. The ethics board at the University of British Columbia exempted the present study from review because it used de-identified and publicly available data. All methods were performed in accordance with the relevant guidelines and regulations.

### Measures

#### Volunteer activity

Respondents were asked: “Have you spent any time in the past 12 months doing volunteer work for religious, educational, health-related, or other charitable organizations?” If they answered yes to this question, respondents were asked how many hours they volunteered: 1–49 h, 50–99 h, 100–199 h, or ≥ 200 h. Results for the binary volunteering variable (no volunteering vs. volunteering) are presented in the main text, while (1) the binary volunteering variable at the 100 h threshold (< 100 h vs. ≥ 100 h), (2) the 4-category ordinal volunteering variable (0 h, 1–49 h, 50–99 h, ≥ 100 h), and (3) the 3-category ordinal volunteering variable (0 h, 1–99 h, ≥ 100 h) are available in the supplementary materials. The 100 h threshold was set based on past findings which suggest that 100 volunteering h/year may be an inflection point for improved health and well-being^4,5,37^. We also collapsed the top two groups (100–199 h, ≥ 200 h) since only ~ 5% of the sample is in the ≥ 200 h group. The 3-category ordinal volunteering variable was added based on prior work assessing volunteering in older adults that distinguished between 0 h (none), 1–99 h (low), and medium or high (100–199 h (medium), and ≥ 200 h (high))^38^.

#### Covariates

We adjusted for a large number of covariates in the pre-baseline wave (t_0_; 2006/2008). Covariates included: sociodemographic factors (age (continuous), gender (male/female), race/ethnicity (White, African-American, Hispanic, Other), marital status (married/not married), income (< $50,000, $50,000–$74,999, $75,000–$99,999, ≥ $100,000), total wealth (based on quintiles of the score distribution for total wealth in this sample), educational attainment (no degree, GED/high school diploma, ≥ college degree), health insurance (yes/no), geographic region (Northeast, Midwest, South, West), personality (openness, conscientiousness, extraversion, agreeableness, neuroticism; continuous), and childhood physical abuse (yes/no)). We adjusted for prior values of all covariates and predictor variables to examine change in each predictor variable. To reduce the possibility of reverse causation, we also adjusted for pre-baseline volunteering. The Supplementary Information (Supplementary Methods) and HRS materials provide further details about each of the variables described in the “Covariates” and “Predictors” sections^35,39,40^.

#### Predictors

We evaluated 61 candidate predictors in the baseline wave (t_1_; 2010/2012). These included measures of: physical health (number of chronic conditions, diabetes, hypertension, stroke, cancer, heart disease, lung disease, arthritis, overweight/obesity, physical functioning limitations, cognitive impairment, chronic pain, self-rated health, eyesight, hearing), health behaviors (heavy drinking, smoking, physical activity, sleep problems), psychological well-being (positive affect, life satisfaction, optimism, purpose in life, mastery, health mastery, financial mastery), psychological distress (depression, depressive symptoms, hopelessness, negative affect, perceived constraints, anxiety, trait anger, state anger, cynical hostility, stressful life events, financial strain, daily discrimination, major discrimination), social factors (loneliness, living with a spouse/partner, frequency of contact with (1) children, (2) other family, and (3) friends, closeness with spouse, number of close (1) children, (2) other family, (3) friends, positive social support from: (1) spouse, (2) children, (3) other family, (4) friends, negative social strain from: (1) spouse, (2) children, (3) other family, (4) friends, religious service attendance, helping friends, neighbors, and relatives, social status ladder ranking, and change in social status ladder ranking), and work (in the labor force).

#### Multiple imputation

All missing exposures, covariates, and outcome variables were imputed using imputation by chained equations, and five datasets were created. This method may be more flexible than other methods of handling missing data^41–43^ and addresses problems that arise from attrition^44–49^.

### Statistical analysis

We used a lagged exposure-wide analytic approach^28^ (see Supplementary Equations) and ran separate models for each exposure. Because volunteering was a binary outcome with a prevalence ≥ 10%, we used generalized linear models (with a log link and Poisson distribution) to individually regress volunteering in the outcome wave (t_2_:2014/2016) on baseline (at t_1_; 2010/2012) candidate predictors. All continuous predictors were standardized (mean = 0, standard deviation = 1) so that their effect sizes could be interpreted as a standard deviation change in the exposure variable. For categorical exposures, the effect estimate corresponds to associations between the exposures at baseline (t_1_; 2010/2012) and volunteering in the outcome wave (t_2_:2014/2016), conditional on the exposures in the pre-baseline wave (t_0_; 2006/2008). We marked multiple p-value cutoffs (including Bonferroni-corrected) in our tables as multiple testing practices vary widely and are continuously evolving and also, perhaps preferably, gave exact confidence intervals^50,51^.

#### Additional analyses

We conducted several additional analyses. First, we conducted E-value analyses to assess the minimum strength of unmeasured confounding associations on the risk ratio scale (with both the exposure and the outcome) needed to explain away the association between the exposure and outcome so as to evaluate the robustness of our results to potential unmeasured confounding^52^. Second, we repeated these analyses only including the subpopulation of people who volunteered at baseline (Supplementary Table 3). This identified what factors predict continued volunteering, four years later, for people who were already volunteering at baseline. Third, we repeated these analyses only including the subpopulation of people who did not volunteer at baseline (Supplementary Table 4). This identified what factors predict volunteering, four years later, for people who did not volunteer at baseline. Fourth, we repeated all analyses using the 100 h threshold binary volunteering variable (Supplementary Tables 5–7). Fifth, we repeated all analyses using the 4-category ordinal volunteering variable (Supplementary Tables 8–10). Sixth, we repeated all analyses using the 3-category ordinal volunteering variable (Supplementary Tables 11–13). Finally, we re-analyzed all models using only complete-cases to assess the impact of multiple imputation on results (Supplementary Table 14).

All HRS data is publicly available (https://hrs.isr.umich.edu/data-products) and code is available upon request.

## Results

In the pre-baseline wave (t_0_; 2006/2008), participants were on average 69 years old (SD = 10), predominantly women (58%), and married (62%). Table 1 provides the distribution of covariates in the pre-baseline wave. Supplementary Table 1 describes the *changes* in volunteering from the pre-baseline wave (t_0_) to the outcome wave (t_2_), and Supplementary Table 2 shows the changes in candidate predictors from the pre-baseline wave (t_0_) to the baseline wave (t_1_).

**Table 1:** Characteristics of participants at pre-baseline (N = 13,755)^a,b,c^.

Participant characteristics	Pre-baseline: non-volunteer N = 8,928		Pre-baseline: volunteer N = 4,827	
	No. (%)	Mean (SD)	No. (%)	Mean (SD)
**Sociodemographic factors**				
Age (years; range: 52–104)		69.7 (9.9)		68.3 (9.0)
Female (%)	5116 (57.3)		2912 (60.3)	
Race/ethnicity (%)				
White	6680 (74.8)		3950 (81.8)	
Black	1158 (13.0)		601 (12.5)	
Hispanic	893 (10.0)		194 (4.0)	
Other	196 (2.2)		82 (1.7)	
Married (%)	5234 (58.6)		3346 (69.3)	
Annual household income (%)				
< $50,000	5939 (66.5)		2411 (50.0)	
$50,000–$74,999	1274 (14.3)		844 (17.5)	
$75,000–$99,999	632 (7.1)		518 (10.7)	
≥ $100,000	1083 (12.1)		1054 (21.8)	
Total wealth (%)				
1st Quintile	2167 (24.3)		584 (12.1)	
2nd Quintile	1967 (22.0)		788 (16.3)	
3rd Quintile	1748 (19.6)		1001 (20.7)	
4th Quintile	1589 (17.8)		1160 (24.0)	
5th Quintile	1457 (16.3)		1294 (26.8)	
Education (%)				
< High school	2267 (25.4)		444 (9.2)	
High school	4940 (55.4)		2565 (53.3)	
≥ College	1706 (19.1)		1805 (37.5)	
Employment (%)				
In labor force	2802 (31.4)		1976 (40.9)	
Health insurance (%)	8488 (95.1)		4680 (97.0)	
Geographic region (%)				
Northeast	1421 (16.0)		667 (13.8)	
Midwest	2171 (24.4)		1420 (29.4)	
South	3643 (40.9)		1848 (38.3)	
West	1674 (18.8)		888 (18.4)	
Childhood abuse (%)	572 (6.5)		277 (5.8)	
**Physical health**				
Number of physical conditions (range: 0–8)		2.7 (1.5)		2.4 (1.4)
Diabetes (%)	1947 (21.8)		778 (16.1)	
Hypertension (%)	5233 (58.7)		2608 (54.1)	
Stroke (%)	853 (9.6)		256 (5.3)	
Cancer (%)	1421 (16.0)		666 (13.8)	
Heart disease (%)	2346 (26.3)		1008 (20.9)	
Lung disease (%)	996 (11.2)		305 (6.3)	
Arthritis (%)	5501 (61.7)		2794 (57.9)	
Overweight/obesity (%)	6127 (69.6)		3355 (70.1)	
Physical functioning limitations (%)	2600 (29.1)		724 (15.0)	
Cognitive impairment (%)	2175 (24.9)		524 (10.9)	
Chronic pain (%)	3351 (37.5)		1398 (29.0)	
Self-rated health (range: 1–5)		3.0 (1.1)		3.5 (1.0)
Hearing (range: 1–5)		3.2 (1.1)		3.4 (1.0)
Eyesight (range: 1–6)		4.1 (1.0)		4.4 (0.9)
**Health behaviors**				
Heavy drinking (%)	550 (7.4)		241 (6.1)	
Smoking (%)	1413 (15.9)		309 (6.5)	
Frequent physical activity (%)	5880 (65.9)		3975 (82.4)	
Sleep problems (%)	2042 (44.0)		1008 (38.6)	
**Psychological well-being**				
Positive affect (range: 1–5)		3.5 (0.8)		3.8 (0.7)
Life satisfaction (range: 1–7)		4.9 (1.5)		5.3 (1.4)
Optimism (range: 1–6)		4.3 (1.0)		4.7 (0.9)
Purpose in life (range: 1–6)		4.4 (1.0)		4.8 (0.8)
Mastery (range: 1–6)		4.7 (1.1)		4.9 (1.0)
Health mastery (range: 0–10)		7.1 (2.5)		7.5 (2.1)
Financial mastery (range: 0–10)		7.2 (2.8)		7.6 (2.3)
**Psychological distress**				
Depression (%)	1509 (17.3)		369 (7.7)	
Depressive symptoms (range: 0–8)		1.7 (2.1)		0.9 (1.6)
Hopelessness (range: 1–6)		2.6 (1.3)		2.0 (1.1)
Negative affect (range: 1–5)		1.7 (0.7)		1.6 (0.5)
Perceived constraints (range: 1–6)		2.4 (1.3)		1.9 (1.0)
Anxiety (range: 1–4)		1.6 (0.6)		1.5 (0.5)
Trait anger (range: 1–4)		2.2 (0.7)		2.1 (0.7)
State anger (range: 1–4)		1.5 (0.5)		1.4 (0.4)
Cynical hostility (range: 1–6)		3.1 (1.2)		2.7 (1.1)
Stressful life events (range: 0–5)		0.2 (0.6)		0.2 (0.5)
Financial strain (range: 1–5)		2.0 (1.0)		1.8 (0.9)
Daily discrimination (range: 1–6)		1.6 (0.8)		1.6 (0.7)
Major discrimination (range: 0–6)		0.4 (0.9)		0.5 (0.9)
**Social factors**				
Living with spouse/partner (%)	5400 (62.6)		3388 (71.6)	
Contact children (%)				
< Every few months	1312 (15.1)		528 (11.2)	
1-2x/month	963 (11.1)		547 (11.6)	
1-2x/week	2508 (28.9)		1622 (34.3)	
≥ 3x/week	3884 (44.8)		2028 (42.9)	
Contact other family (%)				
< Every few months	2184 (25.1)		1089 (23.0)	
1-2x/month	1920 (22.1)		1198 (25.3)	
1-2x/week	2304 (26.5)		1370 (29.0)	
≥ 3x/week	2278 (26.2)		1076 (22.7)	
Contact friends (%)				
< Every few months	1889 (21.6)		375 (7.9)	
1-2x/month	1634 (18.7)		846 (17.8)	
1-2x/week	2845 (32.6)		1959 (41.1)	
≥ 3x/week	2363 (27.1)		1584 (33.3)	
Loneliness (range: 1–3)		1.5 (0.6)		1.4 (0.5)
Closeness with spouse (range: 1–4)		3.4 (0.8)		3.5 (0.7)
Number of close children		2.9 (4.1)		2.7 (2.9)
Number of close other family		3.9 (5.8)		3.9 (5.0)
Number of close friends		4.0 (5.6)		5.4 (6.5)
Positive social support from spouse (range: 1–4)		3.4 (0.7)		3.5 (0.6)
Positive social support from children (range: 1–4)		3.3 (0.7)		3.3 (0.7)
Positive social support from other family (range: 1–4)		2.9 (0.9)		2.9 (0.8)
Positive social support from friends (range: 1–4)		3.0 (0.8)		3.1 (0.7)
Social strain from spouse (range: 1–4)		2.0 (0.7)		1.9 (0.6)
Social strain from children (range: 1–4)		1.7 (0.7)		1.7 (0.6)
Social strain from other family (range: 1–4)		1.6 (0.6)		1.6 (0.6)
Social strain from friends (range: 1–4)		1.8 (0.4)		1.9 (0.4)
Religious service attendance (%)				
Not at all	2994 (33.6)		457 (9.5)	
< 1x/week	3136 (35.2)		1159 (24.0)	
≥ 1x/week	2792 (31.3)		3208 (66.5)	
Helping friends/neighbors/relatives (%)				
0 h/year	5221 (58.6)		1400 (29.1)	
1–49 h/year	1806 (20.3)		1412 (29.4)	
50–99 h/year	861 (9.7)		974 (20.3)	
100–199 h/year	571 (6.4)		618 (12.9)	
≥ 200 h/year	450 (5.1)		406 (8.4)	
Social status ladder (range: 1–10)		6.3 (1.8)		6.9 (1.6)
Change in social status ladder (%)				
Moved down	880 (10.3)		385 (8.2)	
No change	6598 (77.3)		3686 (78.7)	
Moved up	1056 (12.4)		614 (13.1)	
**Personality**				
Openness (range: 1–4)		2.9 (0.6)		3.0 (0.5)
Conscientiousness (range: 1–4)		3.3 (0.5)		3.4 (0.4)
Extraversion (range: 1–4)		3.1 (0.6)		3.3 (0.5)
Agreeableness (range: 1–4)		3.5 (0.5)		3.6 (0.4)
Neuroticism (range: 1–4)		2.1 (0.6)		1.9 (0.6)

^a^This table was created based on non-imputed data.

^b^All variables in Table 1 were used as covariates, and assessed in the pre-baseline wave (t_0_; 2006/2008).

^c^The percentages in some sections may not add up to 100% due to rounding.

Those who participated in frequent (≥ 1x/week) physical activity (at baseline t_1_; 2010/2012) had an 18% increased likelihood of volunteering (95% CI 1.08, 1.29), and those who reported smoking at baseline had a 21% decreased likelihood of volunteering (95% CI 0.63, 0.98) four years later (Table 2). However, there was little evidence of associations between other health behaviors (e.g., heavy drinking and sleep problems) and subsequent volunteering.

**Table 2:** Candidate predictors of volunteering (volunteering vs. no volunteering) (Health and Retirement Study [HRS]: N = 13,771)^a,b,c^.

Candidate predictor	Risk ratio	95% CI
**Health behaviors**		
Frequent physical activity	1.18	1.08, 1.29***
Smoking	0.79	0.63, 0.98*
Heavy drinking	1.07	0.90, 1.28
Sleep problems	0.95	0.88, 1.03
**Physical health**		
Number of physical conditions	0.97	0.90, 1.05
Diabetes	0.92	0.79, 1.07
Hypertension	1.04	0.88, 1.22
Stroke	0.78	0.62, 0.99*
Cancer	1.02	0.87, 1.21
Heart disease	0.96	0.82, 1.14
Lung disease	0.84	0.62, 1.14
Arthritis	1.05	0.91, 1.20
Overweight/obese	1.00	0.90, 1.12
Physical functioning limitations	0.79	0.70, 0.89***
Cognitive impairment	0.77	0.68, 0.86***
Chronic pain	0.96	0.86, 1.07
Self-rated health	1.07	1.02, 1.13**
Hearing	1.01	0.95, 1.07
Eyesight	1.04	0.99, 1.09
**Psychological well-being**		
Positive affect	1.12	1.06, 1.17***
Life satisfaction	1.07	1.01, 1.13*
Optimism	1.03	0.98, 1.08
Purpose in life	1.10	1.04, 1.16**
Mastery	1.05	1.00, 1.10*
Health mastery	1.04	0.99, 1.09
Financial mastery	1.03	0.98, 1.07
**Psychological distress**		
Depression	0.90	0.77, 1.05
Depressive symptoms	0.93	0.88, 0.98*
Hopelessness	0.94	0.89, 0.99*
Negative affect	0.96	0.91, 1.02
Constraints	0.90	0.85, 0.96**
Anxiety	0.94	0.89, 0.99*
Trait anger	0.98	0.93, 1.02
State anger	0.94	0.89, 1.00*
Cynical hostility	0.97	0.92, 1.01
Stressful life events	1.01	0.98, 1.05
Financial strain	1.00	0.95, 1.06
Daily discrimination	0.99	0.95, 1.04
Major discrimination	1.04	0.99, 1.08
**Social factors**		
Living with spouse/partner	0.91	0.79, 1.04
Contact children		
< Every few months	Reference	Reference
1-2x/month	1.07	0.88, 1.29
1-2x/week	1.15	0.98, 1.36
≥ 3x/week	1.08	0.91, 1.28
Contact other family		
< Every few months	Reference	Reference
1-2x/month	1.03	0.92, 1.15
1-2x/week	1.01	0.91, 1.13
≥ 3x/week	1.03	0.92, 1.15
Contact friends		
< Every few months	Reference	Reference
1-2x/month	1.28	1.11, 1.48**
1-2x/week	1.38	1.22, 1.57***
≥ 3x/week	1.46	1.27, 1.67***
Loneliness	0.99	0.94, 1.05
Closeness with spouse	0.98	0.88, 1.08
Number of close children	0.99	0.95, 1.03
Number of close other family	1.01	0.98, 1.05
Number of close friends	1.03	0.99, 1.06
Positive social support from spouse	1.04	0.99, 1.10
Positive social support from children	1.03	0.98, 1.08
Positive social support from other family	0.99	0.95, 1.03
Positive social support from friends	1.07	1.03, 1.12**
Social strain from spouse	0.96	0.91, 1.02
Social strain from children	0.96	0.92, 1.01
Social strain from other family	1.01	0.96, 1.06
Social strain from friends	1.04	1.00, 1.09*
Religious service attendance		
Not at all	Reference	Reference
< 1x/week	1.50	1.30, 1.74***
≥ 1x/week	2.30	1.97, 2.68***
Helping friends/neighbors/relatives		
0 h/year	Reference	Reference
1–49 h/year	1.21	1.11, 1.32***
50–99 h/year	1.33	1.20, 1.49***
100–199 h/year	1.31	1.14, 1.50***
≥ 200 h/year	1.30	1.12, 1.51***
Social status ladder	1.04	0.99, 1.10
Change in social status ladder		
Moved down	Reference	Reference
No change	0.99	0.89, 1.10
Moved up	1.00	0.86, 1.16
**Work**		
In labor force	0.93	0.84, 1.02

CI, confidence interval; RR, risk ratio.

*P < .05 before Bonferroni correction; **P < .01 before Bonferroni correction; ***P < .05 after Bonferroni correction (the *P* value cutoff for Bonferroni correction is *P* = .05/61 predictors = *P* < .00081968).

^a^The analytic sample was restricted to those who had participated in the pre-baseline wave (2006 or 2008). Multiple imputation was performed to impute missing data on the exposures, covariates, and outcome. Candidate antecedents were assessed, one at a time, in wave 2 (2010/2012), and the outcome (volunteering) was assessed in wave 3 (2014/2016). The following covariates were controlled for at wave 1 (2006/2008): sociodemographic characteristics (age, sex, race/ethnicity, marital status, income, total wealth, level of education, health insurance, geographic region), childhood abuse, personality factors (openness, conscientiousness, extraversion, agreeableness, neuroticism), and all of the predictor variables, including: health behaviors (physical activity, smoking, heavy drinking, sleep problems), physical health (heart disease, cancer, stroke, arthritis, hypertension, overweight/obese, diabetes, lung disease, chronic pain, hearing, eyesight, self-rated health, physical functioning limitations, cognitive impairment), social factors (live with spouse, frequency of contact with children, frequency of contact with other family, frequency of contact with friends, loneliness, closeness with spouse, number of close children, number of close other family, number of close friends, positive social support from spouse, positive social support from children, positive social support from friends, positive social support from other family, social strain from spouse, social strain from children, social strain from other family, social strain from friends, religious service attendance, volunteering, helping friends/neighbors/relatives, perceived social status, change in perceived social status), psychological well-being factors (life satisfaction, positive affect, purpose in life, optimism, health mastery, financial mastery, mastery), psychological distress (depressive symptoms, hopelessness, negative affect, constraints, anxiety, trait anger, state anger, daily discrimination, major discrimination, cynical hostility, stressful life events, financial strain), and work (in labor force).

^b^All continuous candidate antecedents were standardized (mean = 0; standard deviation = 1).

^c^An exposure-wide analytic approach was used, and a separate model for each exposure was run. Because volunteering was a binary outcome with a prevalence of ≥ 10%, we ran a generalized linear model with a log link and Poisson distribution to estimate a RR.

Among physical health factors, those who reported stroke (at baseline t_1_; 2010/2012) were 22% less likely to volunteer (95% CI 0.62, 0.99), those with physical functioning limitations were 21% less likely to volunteer (95% CI 0.70, 0.89), and those with cognitive impairment were 23% less likely to volunteer (95% CI 0.68, 0.86) four years later. Further, for each standard deviation increase in self-rated health, participants were 7% more likely to volunteer (95% CI 1.02, 1.13). However, there was little evidence of associations between other physical health indicators (e.g., arthritis) and subsequent volunteering.

Among psychological factors, participants were more likely to volunteer for each standard deviation increase in positive affect (RR = 1.12, 95% CI 1.06, 1.17), life satisfaction (RR = 1.07, 95% CI 1.01, 1.13), purpose in life (RR = 1.10, 95% CI 1.04, 1.16), and mastery (RR = 1.05, 95% CI 1.00, 1.10) four years later. Further, participants were less likely to volunteer for every standard deviation increase in depressive symptoms (RR = 0.93, 95% CI 0.88, 0.98), hopelessness (RR = 0.94, 95% CI 0.89, 0.99), constraints (RR = 0.90, 95% CI 0.85, 0.96), anxiety (RR = 0.94, 95% CI 0.89, 0.99), and state anger (RR = 0.94, 95% CI 0.89, 1.00). There was little evidence of associations between other psychological factors (e.g., financial strain) and subsequent volunteering.

For social factors, participants who were in greater contact with friends (e.g., ≥ 3x/week) were 46% more likely to volunteer (95% CI 1.27, 1.67) four years later. For every standard deviation increase in positive social support from friends and negative social strain from friends, participants were 7% (95% CI 1.03, 1.12) and 4% (95% CI 1.00, 1.09) more likely to volunteer, respectively. Participants who frequently attended religious services (≥ 1x/week) were 130% more likely to volunteer (95% CI 1.97, 2.68), and participants who spent time helping friends, neighbors, and relatives (e.g., ≥ 200 h/year) were 30% more likely to volunteer (95% CI 1.12, 1.51). There was little evidence of associations between other social factors (e.g., loneliness) and subsequent volunteering. Employment status was also not associated with subsequent volunteering.

### Additional analyses

We conducted several additional analyses. First, E-values suggested that many of the observed associations were moderately robust to unmeasured confounding (Table 3). For example, for cognitive impairment, an unmeasured confounder associated with both volunteering and cognitive impairment by risk ratios of 1.94 each (above and beyond the covariates already adjusted for) could explain away the association, but weaker joint confounding associations could not. Further, to shift the CI to include the null, an unmeasured confounder associated with both volunteering and cognitive impairment by risk ratios of 1.58 could suffice, but weaker joint confounding associations could not. Second, results were different when comparing candidate predictors for the subpopulations of people who volunteered at baseline vs. those who did not volunteer at baseline (Supplementary Tables 3–4). Third, results were also different comparing results when using the 100 h threshold binary volunteering variable, amongst the entire sample, only people who volunteered regularly at baseline, and only people who did not volunteer regularly at baseline, (Supplementary Tables 5–7). Fourth, results with the 4-category ordinal coding of the volunteering variable (Supplementary Tables 8–10) and the 3-category ordinal coding (Supplementary Tables 11–13) show associations between candidate predictors and subsequent volunteering on an ordinal scale. Fifth, complete-case analyses showed similar results to those from the main imputed analyses (Supplementary Table 14).

**Table 3:** Robustness to unmeasured confounding (E-values) for the associations between candidate antecedents and subsequent volunteering (N = 13,771)^a^.

	Effect estimate^b^	Confidence interval limit^c^
**Health behaviors**		
Frequent physical activity	1.64	1.37
Smoking	1.86	1.15
Heavy drinking	1.34	1.00
Sleep problems	1.29	1.00
**Physical health**		
Number of physical conditions	1.20	1.00
Diabetes	1.39	1.00
Hypertension	1.23	1.00
Stroke	1.87	1.13
Cancer	1.18	1.00
Heart disease	1.24	1.00
Lung disease	1.67	1.00
Arthritis	1.27	1.00
Overweight/obese	1.07	1.00
Physical functioning limitations	1.85	1.51
Cognitive impairment	1.94	1.58
Chronic pain	1.26	1.00
Self-rated health	1.35	1.15
Hearing	1.10	1.00
Eyesight	1.24	1.00
**Psychological well-being**		
Positive affect	1.48	1.32
Life satisfaction	1.33	1.11
Optimism	1.20	1.00
Purpose in life	1.42	1.24
Mastery	1.27	1.02
Health mastery	1.23	1.00
Financial mastery	1.19	1.00
**Psychological distress**		
Depression	1.47	1.00
Depressive symptoms	1.37	1.15
Hopelessness	1.33	1.12
Negative affect	1.23	1.00
Constraints	1.45	1.25
Anxiety	1.33	1.13
Trait Anger	1.18	1.00
State Anger	1.31	1.05
Cynical hostility	1.22	1.00
Stressful life events	1.14	1.00
Financial strain	1.03	1.00
Daily discrimination	1.11	1.00
Major discrimination	1.23	1.00
**Social factors**		
Living with spouse/partner	1.44	1.00
Contact children		
< Every few months	Reference	Reference
1-2x/month	1.33	1.00
1-2x/week	1.57	1.00
> 3x/week	1.37	1.00
Contact other family		
< Every few months	Reference	Reference
1-2x/month	1.21	1.00
1-2x/week	1.14	1.00
>3x/week	1.20	1.00
Contact friends		
< Every few months	Reference	Reference
1-2x/month	1.88	1.46
1-2x/week	2.11	1.73
> 3x/week	2.27	1.86
Loneliness	1.09	1.00
Closeness with spouse	1.19	1.00
Number of close children	1.09	1.00
Number of close other family	1.14	1.00
Number of close friends	1.20	1.00
Positive social support from spouse	1.24	1.00
Positive social support from children	1.20	1.00
Positive social support from other family	1.11	1.00
Positive social support from friends	1.35	1.19
Social strain from spouse	1.25	1.00
Social strain from children	1.24	1.00
Social strain from other family	1.11	1.00
Social strain from friends	1.26	1.05
Religious service attendance		
Not at all	Reference	Reference
< 1x/week	2.37	1.92
> 1x/week	4.02	3.36
Helping friends/neighbors/relatives		
0 h/year	Reference	Reference
1–49 h/year	1.72	1.46
50–99 h/year	2.00	1.69
100–199 h/year	1.95	1.55
≥ 200 h/year	1.93	1.50
Social status ladder	1.26	1.00
Change in social status ladder		
Moved down	Reference	Reference
No change	1.10	1.00
Moved up	1.04	1.00
**Work**		
In labor force	1.36	1.00

^a^See VanderWeele and Ding^52^ for the formula for calculating E-values.

^b^The E-values for effect estimates are the minimum strength of association on the risk ratio scale that an unmeasured confounder would need to have with both the exposure and the outcome to fully explain away the observed association between the exposure and outcome, conditional on the measured covariates.

^c^The E-values for the limit of the 95% confidence interval (CI) closest to the null denote the minimum strength of association on the risk ratio scale that an unmeasured confounder would need to have with both the exposure and the outcome to shift the confidence interval to include the null value, conditional on the measured covariates.

## Discussion

In a large, longitudinal, and national sample of U.S. adults aged > 50, we examined the associations between *changes* in 61 candidate predictors (across physical health, health behaviors, and psychosocial factors) and subsequent volunteering. Some health behaviors (e.g., frequent physical activity), physical health conditions (e.g., physical functioning limitations), psychological well-being (e.g., purpose in life), psychological distress (e.g., constraints), and social factors (e.g., contact with friends) were associated with volunteering four years later. However, there was little evidence of associations between other health behaviors (e.g., sleep problems), physical health conditions (e.g., heart disease), psychosocial factors (e.g., loneliness), employment (e.g., work), and subsequent volunteering. Specifically, the candidate antecedents of greatest predictive importance included physical activity, smoking, stroke, physical functioning limitations, cognitive impairment, sense of purpose in life, positive affect, contact with friends, religious service attendance, and helping friends, neighbors, and relatives, with religious service attendance and contact with friends having the largest effect sizes. It appears that regardless of an individual’s psychological distress (e.g., depression), fostering positive psychological factors (purpose in life) might still increase subsequent volunteering (and vice versa), and likewise regardless of loneliness, fostering greater social engagement might increase volunteering^53^.

Our findings converge with previous studies that observed how increased physical activity^16^, less functional limitations^17^, less cognitive impairment^18^, and increased religious service attendance^17^ were positively associated with volunteering. The latter finding, given it has the largest effect size and is consistent with a large prior body of research^17^, may be of special interest insofar as religious communities represent an important social setting for volunteer mobilization and understanding why this is the case would offer many lessons for other social institutions that might be mobilized to expand volunteering^54,55^. While religious service attendance does provide an additional context in which people can volunteer, the correlation between religious service attendance and volunteering was lower than some might expect. In our study, among people who regularly attended religious services in the baseline wave (≥ 1x/week), only 45% engaged in volunteering in the outcome wave, with fewer than 20% volunteering ≥ 100 h/year. In prior work, there is reciprocity between religiosity and volunteering, but the association of religious volunteering on religiosity is stronger than the other way around^56^. Further, religious service attendance and secular volunteering are reciprocally related, but the reciprocal relation between them is weaker than that of religious volunteering and religious service attendance^56^.

Our findings further converged with previous research that has observed that number of comorbidities is not associated with volunteering^23^. However, our findings diverge from prior work which observed that other factors (e.g., larger social network size)^17^ are associated with increased volunteering. There are many potential reasons for these discrepancies, including differences in: (1) study design (e.g., different follow-up periods), (2) sample composition, (3) measurement and categorization of exposure (e.g., we assessed *changes* in predictors) and outcome variables (e.g., measuring intention to volunteer vs. actual volunteering), and (4) covariate measurement (we used more extensive covariate adjustment than most previous studies). For example, while previous studies have observed that people with a higher perceived ease of volunteering have increased intentions of volunteering^19–22^, many of these studies are about *intention* or *motivation* to volunteer, rather than actual volunteering, which may explain discrepancies in our findings. Further, prior studies have observed that both increased depression and decreased depressive symptoms are associated with increased volunteering^24–26^. While depressive symptoms were associated with a decreased risk of volunteering in our study, depressive symptoms may both decrease volunteering (due to depressive symptoms acting as a barrier to continued volunteering) and increase volunteering (as older adults attempt to compensate for an increased risk of depression derived from role losses and functional decline by volunteering)^24,26^, which may explain our null results for depression.

These diverging results also highlight the importance of several future directions. First, future studies should consider important social structural moderators of the associations between candidate antecedents and volunteering. Prior research has shown that volunteering rates differ across key social structural moderators (e.g., age, race, gender, country, social class, education)^11,15,57^. Women are slightly more likely to volunteer than men in North America^15^. Given the importance of purpose in life in predicting volunteering in our study, and the social factors that encourage women in the United States to both develop a stronger sense of purpose and perform more altruistic service overall (including for family and friends and not limited to formal volunteering), this finding is not surprising^58^. However, further research is needed as these findings are often mixed or ambiguous and variation is apparent across nations (e.g., volunteering rates based on gender may differ based on life stage, with women volunteering more than men at earlier stages in life, and this pattern reversing among older adults; women volunteer more than men in some countries and less than men in others)^15^. Second, there are many other factors that might predict volunteering that we could not assess in the HRS dataset (e.g., various motivational factors)^11,59,60^. For instance, there is evidence that altruism can potentially be intervened upon^61^ which is one potential way we could increase volunteering. Further, there are nuances within our list of predictors (e.g., types of social contact including virtual methods that may be more relevant during this time of COVID-19)^62^ which were not assessed in the HRS dataset. Third, future studies may benefit from assessing predictors of different types of volunteering (e.g., intergenerational volunteering^63,64^, grandparenting roles^65^, etc.) since different modes of volunteering may differentially influence well-being on an individual level and have different societal implications^66^. Indeed, associations between some predictors (e.g., religious service attendance) and volunteering may differ based on the type of volunteering (e.g., religious or secular) being done^56^. Fourth, our volunteering measure contained broad categories of the number of hours spent volunteering and future studies should investigate more nuanced volunteering categories. For older and mostly retired adults, the 100 h threshold might not be as relevant as it is for still-working adults (e.g., older adults may benefit from 4 h per week, rather than 2)^5^. Fifth, future studies should consider mechanistic pathways that explain associations between key predictors in our study (e.g., frequent physical activity) and subsequent volunteering to facilitate increased volunteering. Investigating these mechanistic pathways may explain why some relationship types (e.g., contact with friends) are associated with subsequent volunteering while others (e.g., contact with children) are not. Sixth, examining predictors of volunteering with consideration to relevant life stage transitions and major life events (e.g., changes to caregiver status^67,68^ or employment^25,69^ (e.g., retirement, part-time work, etc.)) may inform volunteer engagement program designs by incorporating time-, situational-, or life event-based approaches to increasing volunteering^11^.

Although there was some variability in the findings reported in the Supplementary Information, which included alternative ordinal outcome measures and limiting the sample to either those with previous volunteer experience or none at all, several variables predicted volunteering across all models. Religious service attendance was perhaps the most consistent. This variable predicted increased volunteering for both previous volunteers and non-volunteers alike, a finding that further underscores the importance of religious settings as a potential site for the study of the social organization of benevolent service to others, and therefore a strategic setting for the advancement of the emerging science of well-doing^70^. Our supplemental analyses also highlight the role of friendship networks in predicting volunteering. Serving others is perhaps most satisfying and positively reinforcing when done in the company of friends, even for those with no previous history of volunteering. Notably, some objective indicators of social contact (e.g., contact with friends) were associated with subsequent volunteering, while some subjective indicators (loneliness) were not. Loneliness is the *subjective* perception of feeling socially disconnected^71^, whereas social isolation is the *objective* lack of social interactions (e.g., smaller social network)^72^. Objective and subjective factors may operate through different mechanisms to influence subsequent volunteering engagement. While frequent contact with friends might encourage joining in on group activities (such as volunteering), the experience of loneliness might not directly predict future volunteering. Perhaps, increased confidence in social skill development may be needed to encourage volunteering in those with high loneliness. However, prior work has found that volunteering is associated with subsequently decreased loneliness^37^, suggesting that while increased loneliness might not lead to volunteering engagement, volunteering may decrease subsequent loneliness. More generally, there is great value in understanding the factors that help shape whether an individual will shift from not volunteering at all to volunteering at a low level, especially if this indicates the beginning of a longer-term trajectory that might result in that person eventually crossing the 100 h/year threshold^4,5^. But any increase in volunteering is potentially beneficial to those being served, regardless of whether the volunteer experiences greater health or well-being. Supplementary Tables 8–10 reveal a broader range of predictors, and therefore more opportunities for social policy interventions to increase volunteering, than we found in our primary analysis. Consideration of these results also permit policy makers to target interventions towards those who have (or have not) had previous volunteering experience.

There is evidence for bidirectional associations between volunteering and health and well-being factors. Many prior studies have assessed how volunteering is associated with subsequent health and well-being outcomes. Observational studies show that volunteering is associated with reduced risk of several chronic conditions (e.g., cognitive impairment, cardiovascular disease)^73,74^, and mortality^37,60,75–80^. Volunteering has been repeatedly associated with well-being^3^, and several mechanisms are hypothesized to underlie the health benefits of volunteering, including the cultivation of psychological well-being (e.g., positive affect, purpose in life, self-efficacy, and reduced depression)^37,75,81–84^ and social well-being (e.g., reduced loneliness and increased frequency of contact with friends)^37^. In turn, these assets might promote healthier behaviors (e.g., increased: use of preventive healthcare services and physical activity)^37,85^ and better biological functioning (e.g., lower: blood pressure and inflammation)^86,87^. Results from experimental volunteering studies generally converge with results from observational studies (e.g., healthier: physical health (physical function, cognitive/neural function)^88–92^, biological functioning (healthier inflammation- and cholesterol-levels)^93,94^, health behaviors (more physical activity)^90,95–97^, and psychosocial health (higher social integration, lower depression))^88,90^. While these prior studies have been interested in how volunteering is associated with subsequent health and well-being outcomes, our study assessed how health and well-being predictors were associated with subsequent volunteering. Prior work in the HRS, using an outcome-wide analytic approach, assessed how changes in volunteering (between t_0_: 2006/2008 and t_1_: 2010/2012) were associated with 34 indicators of physical-, behavioral-, and psychosocial health and well-being four years later^37^. They observed that volunteering was associated with improved physical- (e.g., reduced risk of physical functioning limitations), behavioral- (e.g., higher physical activity), and psychosocial- (e.g., lower loneliness) health and well-being outcomes. Conversely, we assessed how changes in indicators of physical-, behavioral-, and psychosocial-factors (between t_0_: 2006/2008 and t_1_: 2010/2012) were associated with subsequent volunteering. Some key factors associated with volunteering in the previously mentioned outcome-wide study (e.g., physical functioning limitations, physical activity) were also predictors of increased subsequent volunteering, suggesting potential bidirectional associations between some indicators of health and well-being and volunteering. Other studies have found further evidence of potential bidirectional associations between volunteering and health and well-being factors. For example, net of cognitive functioning selection into volunteering, volunteering has been associated with benefits to cognitive function^38^. Future research should further investigate these bi-directional associations.

Our study had several limitations. First, many physical health outcomes, health behaviors, and our measure of volunteering were self-reported in the HRS and thus these measures are vulnerable to self-report bias. However, study participants were unaware of this study’s hypothesis at the time of the study. Second, there is potentially unmeasured confounding. However, we mitigated concerns of unmeasured confounding with robust covariate adjustment, use of a longitudinal design, and E-value analyses. Third, we were unable to evaluate several other potential predictors of volunteering because they were not included in the HRS dataset (e.g., type of culture (independent vs. interdependent), country, the presence of social contacts who volunteer, motivation to volunteer (e.g., extrinsic motivation from an organization, such as church, work, or school, vs. intrinsic motivation to help others), social determinants of health (e.g., ease of transportation) that might impact volunteer engagement, etc.). But our study had several strengths, including the use of a large, diverse, prospective, and national sample of U.S. adults aged > 50 years.

The age group of our sample (older US adults aged > 50) likely plays an important role in our findings. Some of our findings might be understood through the lens of role theory and the role accumulation hypothesis^98^, which hypothesizes that our roles in society and transitions between them (e.g., retirement) exert a powerful influence on our lives as they provide an underlying architecture of societal expectations and reciprocal obligations which elicit new behavioral demands. Certain predictors (e.g., changes to physical health such as physical functioning limitations), are more salient in older populations, while others (e.g., having children younger than 18 years)^99^ are less important in this age group. Likewise, depending on the life course stage, some social relationships (e.g., friends) may be more important than family, which is consistent with our findings that contact with friends was associated with subsequent volunteering, but contact with other family was not. Major life stage transitions may be more relevant in older populations. For example, the effect of employment on volunteering may be influenced by life stage, such that those experiencing work-retirement transitions may be more involved in volunteering than non-retired populations^100^, and other new roles in later life (e.g., caregiving) may influence later life volunteering^67^. Future work will need to continue to investigate factors at the micro-, meso-, and macro-levels that predict volunteering specifically in older populations, predictors of volunteering across the lifespan, and predictors that may be more or less salient at different points during the life course^34^.

As our nations pause and reevaluate our priorities in light of the widespread change that COVID-19 has caused, our public and our policymakers have a rare opportunity to pursue new courageous directions that can simultaneously alleviate important social problems and also help larger portions of our rapidly aging society experience healthy aging. One promising direction is the enactment of bold policies and civic structures, as well as innovative volunteer program designs, that encourage more volunteering among older adults. Many of our study predictors (e.g., positive psychological well-being factors) may be targeted as potential intervention factors to increase subsequent volunteering. For example, there is evidence of life satisfaction being increased through both individual- and population-level interventions and policies^101–105^. A better understanding of how (and why) religious service attendance is so effective at motivating and sustaining volunteering might help support the role of these institutions and also provide clues for the creation of successful non-religious public policies^17,54^. Our aging population represents one of our nation’s only “increasing natural resources,” and this group possesses the skills, experiences, and desire to remain highly productive and contributing members of society^64^. The purpose of our study was to draw attention to specific physical-, behavioral-, and psychosocial-factors that might increase volunteering in older adults. We aimed to explore a more comprehensive pool of options that future investigators might consider as determinants of volunteering. Our study highlights key intervention targets that volunteering program designers may consider in recruiting older volunteers and designing sustainable volunteering opportunities for older adults. Likewise, innovators in the volunteering industry may further investigate these potential intervention targets in predictive models and engagement growth opportunities. As we consider elevating increased volunteering as an important policy and public health goal, our study highlights key intervention targets that governments, volunteering organizations, and corporations can aim at to increase volunteering rates in our nation. Increasing the volunteer workforce may benefit individuals and caregivers (e.g., increasing the healthspan, promoting aging in place), communities (e.g., increasing social cohesion), and the economy (e.g., benefiting communities and reducing health care costs).

## Supplementary Information

Supplementary Information.

## Data Availability

All HRS data is publicly available (https://hrs.isr.umich.edu/data-products) and we are happy to share syntax with people who contact us for it.

## References

[1] Grimm, R.T. Jr. & Dietz, N. Where are America’s volunteers? A look at America's widespread decline in volunteering in cities and states. *Do Good Institute* https://dogood.umd.edu/research-impact/publications/where-are-americas-volunteers (2018).

[2] Demographics. *Corporation for National and Community Service* https://www.nationalservice.gov/serve/via/demographics.

[3] Burr JA, Mutchler JE, Han SH, Ferraro KF, Carr D (2021). Chapter 19—Volunteering and health in later life. Handbook of Aging and the Social Sciences.

[4] Johnson SS, Post SG (2017). Rx It’s good to be good (G2BG) 2017 commentary: Prescribing volunteerism for health, happiness, resilience, and longevity. Am. J. Health Promot..

[5] Johnson SS (2017). The art of health promotion ideas for improving health outcomes. Am. J. Health Promot..

[6] United Nations, Department of Economic and Social Affairs, Population Division. World Population Ageing 2019 (2020).

[7] Colby, S. L. & Ortman, J. M. Projections of the size and composition of the U.S. population. *US Census Bur.* P25–1143 (2014).

[8] Kubzansky LD (2018). Positive psychological well-being and cardiovascular health promotion. J. Am. Coll. Cardiol..

[9] VanderWeele TJ (2017). On the promotion of human flourishing. Proc. Natl. Acad. Sci..

[10] Carr DC, Fried LP, Rowe JW (2015). Productivity & engagement in an aging America: the role of volunteerism. Daedalus.

[11] Morrow-Howell N (2010). Volunteering in later life: Research frontiers. J. Gerontol. Ser. B.

[12] Appau S, Awaworyi Churchill S (2019). Charity, volunteering type and subjective wellbeing. Volunt. Int. J. Volunt. Nonprofit Organ..

[13] Mohan J, Bennett MR (2019). Community-level impacts of the third sector: does the local distribution of voluntary organizations influence the likelihood of volunteering?. Environ. Plan. Econ. Space.

[14] O’Dea, E., Wister, A. & Canham, S. L. Cultural generativity in perspective: Motivations of older Jewish volunteers. *Ageing Soc.* 1–19 (2021).

[15] Wilson J (2000). Volunteering. Annu. Rev. Sociol..

[16] Jongenelis MI (2020). Factors associated with formal volunteering among retirees. Eur. J. Ageing.

[17] Niebuur J, van Lente L, Liefbroer AC, Steverink N, Smidt N (2018). Determinants of participation in voluntary work: A systematic review and meta-analysis of longitudinal cohort studies. BMC Public Health.

[18] Hosking DE, Anstey KJ (2016). The economics of cognitive impairment: volunteering and cognitive function in the HILDA survey. Gerontology.

[19] Grano C, Lucidi F, Zelli A, Violani C (2008). Motives and determinants of volunteering in older adults: an integrated model. Int. J. Aging Hum. Dev..

[20] Hyde MK, Knowles SR (2013). What predicts Australian university students’ intentions to volunteer their time for community service?. Aust. J. Psychol..

[21] Abdulelah, A., Sallam, A., Safizal, M. & Osman, A. The key drivers of volunteering intention among undergraduate Malaysian students an application of theory of planned behavior. https://www.semanticscholar.org/paper/the-key-drivers-of-volunteering-intention-among-an-abdulelah-sallam/023a5b87eceb9f08852f8afbb96bfe66e0326e64 (2015).

[22] Tiraieyari N, Ricard RM, McLean GN (2019). Factors influencing volunteering in urban agriculture: Implications for recruiting volunteers. Urban For. Urban Green..

[23] Ahn S, Phillips KL, Smith ML, Ory MG (2011). Correlates of volunteering among aging Texans: The roles of health indicators, spirituality, and social engagement. Maturitas.

[24] Li Y, Ferraro KF (2005). Volunteering and depression in later life: Social benefit or selection processes?. J. Health Soc. Behav..

[25] Choi LH (2003). Factors affecting volunteerism among older adults. J. Appl. Gerontol..

[26] Li Y, Ferraro KF (2006). Volunteering in middle and later life: Is health a benefit, barrier or both?. Soc. Forces.

[27] VanderWeele TJ (2021). Can sophisticated study designs with regression analyses of observational data provide causal inferences?. JAMA Psychiat..

[28] VanderWeele TJ, Mathur MB, Chen Y (2020). Outcome-wide longitudinal designs for causal inference: A new template for empirical studies. Stat. Sci..

[29] Ryff CD, Singer B (2009). Understanding healthy aging: Key components and their integration. Handb. Theor. Aging.

[30] Rowe JW, Kahn RL (1987). Human aging: Usual and successful. Science.

[31] Reich JW, Zautra AJ, Hall JS (2010). Handbook of Adult Resilience.

[32] Aldwin, C. M. & Igarashi, H. Successful, optimal, and resilient aging: A psychosocial perspective (2015).

[33] Depp CA, Jeste DV (2006). Definitions and predictors of successful aging: a comprehensive review of larger quantitative studies. Am. J. Geriatr. Psychiatry.

[34] Lu P, Xu C, Shelley M (2021). A state-of-the-art review of the socio-ecological correlates of volunteerism among older adults. Ageing Soc..

[35] Smith, J., Ryan, L., Fisher, G. G., Sonnega, A. & Weir, D. Documentation report core section LB. 72 (2017).

[36] Sonnega A (2014). Cohort profile: The Health and Retirement Study (HRS). Int. J. Epidemiol..

[37] Kim ES, Whillans AV, Lee MT, Chen Y, VanderWeele TJ (2020). Volunteering and subsequent health and well-being in older adults: an outcome-wide longitudinal approach. Am. J. Prev. Med..

[38] Kail BL, Carr DC (2020). More than selection effects: Volunteering is associated with benefits in cognitive functioning. J. Gerontol. Ser. B.

[39] Fisher, G. G., Faul, J. D., Weir, D. R. & Wallace, R. B. Documentation of chronic disease measures in the Health and Retirement Study (HRS/AHEAD) (2005).

[40] Jenkins, K. R., Ofstedal, M. B. & Weir, D. Documentation of health behaviors and risk factors measured in the Health and Retirement Study (HRS/AHEAD) (2008).

[41] Sterne JAC (2009). Multiple imputation for missing data in epidemiological and clinical research: potential and pitfalls. BMJ.

[42] Moons KGM, Donders RART, Stijnen T, Harrell FE (2006). Using the outcome for imputation of missing predictor values was preferred. J. Clin. Epidemiol..

[43] Groenwold RHH, Donders ART, Roes KCB, Harrell FE, Moons KGM (2012). Dealing with missing outcome data in randomized trials and observational studies. Am. J. Epidemiol..

[44] Asendorpf JB, van de Schoot R, Denissen JJA, Hutteman R (2014). Reducing bias due to systematic attrition in longitudinal studies: The benefits of multiple imputation. Int. J. Behav. Dev..

[45] Rawlings AM (2017). Multiple imputation of cognitive performance as a repeatedly measured outcome. Eur. J. Epidemiol..

[46] Cumming JJ, Goldstein H (2016). Handling attrition and non-response in longitudinal data with an application to a study of Australian youth. Longitud. Life Course Stud..

[47] Weuve J (2015). Guidelines for reporting methodological challenges and evaluating potential bias in dementia research. Alzheimers Dement. J. Alzheimers Assoc..

[48] Harel O (2018). Multiple imputation for incomplete data in epidemiologic studies. Am. J. Epidemiol..

[49] van Ginkel JR, Linting M, Rippe RCA, van der Voort A (2020). Rebutting existing misconceptions about multiple imputation as a method for handling missing data. J. Pers. Assess..

[50] Dunn OJ (1961). Multiple comparisons among means. J. Am. Stat. Assoc..

[51] VanderWeele TJ, Mathur MB (2019). Some desirable properties of the Bonferroni correction: is the Bonferroni correction really so bad?. Am. J. Epidemiol..

[52] VanderWeele TJ, Ding P (2017). Sensitivity analysis in observational research: Introducing the E-value. Ann. Intern. Med..

[53] Poulin MJ, Brown SL, Dillard AJ, Smith DM (2013). Giving to others and the association between stress and mortality. Am. J. Public Health..

[54] Lee M, Poloma M, Post S (2013). Heart of Religion: Spiritual Empowerment, Benevolence, and the Experience of God’s Love.

[55] Putnam RD, Campbell DE, Garrett SR (2012). American Grace: How Religion Divides and Unites Us.

[56] Son J, Wilson J (2021). Is there a bidirectional causal relationship between religiosity and volunteering?. J. Sci. Study Relig..

[57] Korndörfer M, Egloff B, Schmukle SC (2015). A large scale test of the effect of social class on prosocial behavior. PLoS ONE.

[58] Xi J (2017). Altruism and existential well-being. Appl. Res. Qual. Life.

[59] Wardell F, Lishman J, Whalley L (2000). Who volunteers?. Br. J. Soc. Work.

[60] Konrath S, Fuhrel-Forbis A, Lou A, Brown S (2012). Motives for volunteering are associated with mortality risk in older adults. Health Psychol. Off. J. Div. Health Psychol. Am. Psychol. Assoc..

[61] Weng HY (2013). Compassion training alters altruism and neural responses to suffering. Psychol. Sci..

[62] Lachance EL (2021). COVID-19 and its impact on volunteering: moving towards virtual volunteering. Leis. Sci..

[63] George DR (2011). Intergenerational volunteering and quality of life: mixed methods evaluation of a randomized control trial involving persons with mild to moderate dementia. Qual. Life Res..

[64] Glass TA (2004). Experience Corps: Design of an intergenerational program to boost social capital and promote the health of an aging society. J. Urban Health Bull. N. Y. Acad. Med..

[65] Bulanda JR, Jendrek MP (2016). Grandparenting roles and volunteer activity. J. Gerontol. Ser. B.

[66] Hui BPH, Ng JCK, Berzaghi E, Cunningham-Amos LA, Kogan A (2020). Rewards of kindness? A meta-analysis of the link between prosociality and well-being. Psychol. Bull..

[67] Choi NG, Burr JA, Mutchler JE, Caro FG (2007). Formal and informal volunteer activity and spousal caregiving among older adults. Res. Aging.

[68] Burr JA, Choi NG, Mutchler JE, Caro FG (2005). Caregiving and volunteering: are private and public helping behaviors linked?. J. Gerontol. Ser. B.

[69] Lengfeld H, Ordemann J (2016). The long shadow of occupation: Volunteering in retirement. Ration. Soc..

[70] Lieder, F., Prentice, M. & Corwin-Renner, E. Understanding and promoting effective well-doing: open questions and emerging approaches. Preprint at https://www.researchgate.net/publication/347468246_Understanding_and_promoting_effective_well-doing_open_questions_and_emerging_approaches (2021).

[71] Perlman D, Peplau LA (1981). Toward a social psychology of loneliness. Pers. Relatsh..

[72] Cacioppo S, Capitanio JP, Cacioppo JT (2014). Toward a neurology of loneliness. Psychol. Bull..

[73] Burr JA, Han SH, Tavares JL (2016). Volunteering and cardiovascular disease risk: Does helping others get “under the skin?”. Gerontologist.

[74] Infurna FJ, Okun MA, Grimm KJ (2016). Volunteering is associated with lower risk of cognitive impairment. J. Am. Geriatr. Soc..

[75] Anderson ND (2014). The benefits associated with volunteering among seniors: A critical review and recommendations for future research. Psychol. Bull..

[76] Poulin MJ (2014). Volunteering predicts health among those who value others: two national studies. Health Psychol. Off. J. Div. Health Psychol. Am. Psychol. Assoc..

[77] Musick MA, Herzog AR, House JS (1999). Volunteering and mortality among older adults: Findings from a national sample. J. Gerontol. B. Psychol. Sci. Soc. Sci..

[78] Oman D, Thoresen CE, McMahon K (1999). Volunteerism and mortality among the community-dwelling elderly. J. Health Psychol..

[79] Luoh M-C, Herzog AR (2002). Individual consequences of volunteer and paid work in old age: Health and mortality. J. Health Soc. Behav..

[80] Sabin EP (1993). Social relationships and mortality among the elderly. J. Appl. Gerontol..

[81] Morrow-Howell N, Hinterlong J, Rozario PA, Tang F (2003). Effects of volunteering on the well-being of older adults. J. Gerontol. B. Psychol. Sci. Soc. Sci..

[82] Müller D, Ziegelmann JP, Simonson J, Tesch-Roemer C, Huxhold O (2014). Volunteering and subjective well-being in later adulthood: Is self-efficacy the key?. Int. J. Dev. Sci..

[83] Aknin, L., Whillans, A. V., Norton, M. I. & Dunn, E. W. Happiness and prosocial behavior: an evaluation of the evidence. In *World Happiness Report*. 66–85 (Sustainable Development Solutions Network, 2019).

[84] Tabassum F, Mohan J, Smith P (2016). Association of volunteering with mental well-being: A lifecourse analysis of a national population-based longitudinal study in the UK. BMJ Open.

[85] Kim ES, Konrath SH (2016). Volunteering is prospectively associated with health care use among older adults. Soc. Sci. Med..

[86] Kim S, Ferraro KF (2014). Do productive activities reduce inflammation in later life? Multiple roles, frequency of activities, and C-reactive protein. Gerontologist.

[87] Sneed RS, Cohen S (2013). A prospective study of volunteerism and hypertension risk in older adults. Psychol. Aging.

[88] Hong SI, Morrow-Howell N (2010). Health outcomes of Experience Corps: A high-commitment volunteer program. Soc. Sci. Med..

[89] Barron JS (2009). Potential for intensive volunteering to promote the health of older adults in fair health. J. Urban Health Bull. N. Y. Acad. Med..

[90] Fried LP (2004). A social model for health promotion for an aging population: initial evidence on the Experience Corps model. J. Urban Health Bull. N. Y. Acad. Med..

[91] Carlson MC (2015). Impact of the Baltimore Experience Corps Trial on cortical and hippocampal volumes. Alzheimers Dement..

[92] Carlson MC (2008). Exploring the effects of an “everyday” activity program on executive function and memory in older adults: Experience Corps®. Gerontologist.

[93] Schreier HC, Schonert-Reichl KA, Chen E (2013). Effect of volunteering on risk factors for cardiovascular disease in adolescents: A randomized controlled trial. JAMA Pediatr..

[94] Nelson-Coffey SK, Fritz MM, Lyubomirsky S, Cole SW (2017). Kindness in the blood: A randomized controlled trial of the gene regulatory impact of prosocial behavior. Psychoneuroendocrinology.

[95] Varma VR (2016). Effect of community volunteering on physical activity: a randomized controlled trial. Am. J. Prev. Med..

[96] Parisi JM (2015). Increases in lifestyle activities as a result of Experience Corps® participation. J. Urban Health Bull. N. Y. Acad. Med..

[97] Tan EJ, Xue Q-L, Li T, Carlson MC, Fried LP (2006). Volunteering: a physical activity intervention for older adults—The Experience Corps program in Baltimore. J. Urban Health Bull. N. Y. Acad. Med..

[98] Thoits PA (2012). Role-identity salience, purpose and meaning in life, and well-being among volunteers. Soc. Psychol. Q..

[99] Bureau of Labor Statistics. Volunteering in the United States, 2006. 12. (2007).

[100] Tang F (2016). Retirement patterns and their relationship to volunteering. Nonprofit Volunt. Sect. Q..

[101] Bolier L (2013). Positive psychology interventions: A meta-analysis of randomized controlled studies. BMC Public Health.

[102] Wiese CW, Kuykendall L, Tay L (2018). Get active? A meta-analysis of leisure-time physical activity and subjective well-being. J. Posit. Psychol..

[103] Stiglitz JE, Fitoussi J-P, Durand M (2018). Beyond GDP: Measuring What Counts for Economic and Social Performance.

[104] World Health Organization. Joint meeting of experts on targets and indicators for health and well-being in Health 2020: Copenhagen, Denmark, 5–7 February 2013. *World Health Organ. Reg. Off. Eur.* (2013).

[105] The Global Council for Happiness and Wellbeing (2019). Global Happiness and Wellbeing Policy Report.

