# Supplementary material for: Identifying pathways to increased volunteering in older US adults
Source: Sci Rep. 2022 Jul 27;12:12825. doi: 10.1038/s41598-022-16912-x (PMC9328015; doi:10.1038/s41598-022-16912-x)
Supplement: Supplementary file 1 — Supplementary Information. [file 41598_2022_16912_MOESM1_ESM.docx]

**Supplementary Information**

**Supplementary Methods.** Assessment of Predictors

**Supplementary Equations.** Proof Illustrating How Adjusting for Pre-Baseline Levels of Exposure Can Help Us Evaluate How “Changes” in Exposure are Associated with Subsequent Volunteering Over Time

**Supplementary Table 1.** Changes in Volunteering from the Pre-Baseline Wave (t_0_) to the Outcome Wave (t_2_) – 4 Category Ordinal Volunteering Variable

**Supplementary Table 2.** Changes in Candidate Predictors from the Pre-Baseline Wave (t_0_) to the Baseline Wave (t_1_)

**Supplementary Table 3.** Candidate Predictors of Volunteering: Among People Who Volunteer at Baseline (Health and Retirement Study [HRS]: N = 4,092)

**Supplementary Table 4.** Candidate Predictors of Volunteering: Among People Who Do Not Volunteer at Baseline (Health and Retirement Study [HRS]: N = 7,357)

**Supplementary Table 5.** Candidate Predictors of Volunteering 100-Hour Threshold (Health and Retirement Study [HRS]: N = 13,771)

**Supplementary Table 6.** Candidate Predictors of Volunteering: Among People Who Volunteer Regularly (>or=100 Hours/Week) at Baseline (Health and Retirement Study [HRS]: N = 1,679)

**Supplementary Table 7.** Candidate Predictors of Volunteering: Among People Who Do Not Volunteer Regularly (<100 Hours/Week) at Baseline (Health and Retirement Study [HRS]: N = 9,770)

**Supplementary Table 8.** Candidate Predictors of Volunteering – Ordinal Volunteering Variable (4 categories) (Health and Retirement Study [HRS]: N = 13,771)

**Supplementary Table 9.** Candidate Predictors of Volunteering – Ordinal Volunteering Variable: Among People Who Volunteer at Baseline (4 categories) (Health and Retirement Study [HRS]: N = 4,092)

**Supplementary Table 10.** Candidate Predictors of Volunteering – Ordinal Volunteering Variable (4 categories): Among People Who Do Not Volunteer at Baseline (Health and Retirement Study [HRS]: N = 7,357)

**Supplementary Table 11.** Candidate Predictors of Volunteering – Ordinal Volunteering Variable (3 categories) (Health and Retirement Study [HRS]: N = 13,771)

**Supplementary Table 12.** Candidate Predictors of Volunteering – Ordinal Volunteering Variable: Among People Who Volunteer at Baseline (3 categories) (Health and Retirement Study [HRS]: N = 4,092)

**Supplementary Table 13.** Candidate Predictors of Volunteering – Ordinal Volunteering Variable (3 categories): Among People Who Do Not Volunteer at Baseline (Health and Retirement Study [HRS]: N = 7,357)

**Supplementary Table 14.** Complete-Case Analyses: Candidate Predictors of Volunteering (Health and Retirement Study [HRS]: N ranged from = 3,030 to 5,463)

**Supplementary Methods**

**Assessment of Predictors**

**Reference Group**

The reference group was the healthiest group for all binary outcomes unless otherwise noted.

**Physical Health­­­­**

*Physical conditions.* Participants self-reported (yes/no) if they were ever told by a healthcare provider that they had the following conditions: 1) diabetes, 2) hypertension, 3) stroke, 4) cancer, 5) heart disease, 6) lung disease, or 7) arthritis. The HRS has demonstrated validity and reliability of self-reported chronic conditions.^1^

*Overweight/obesity*. Body mass index (BMI) was derived from self-reported height and weight. It was calculated as weight/height^2^ (kg/m^2^). A BMI of ≥25 kg/m^2^ was considered as overweight/obese.^2^

*Number of chronic conditions.* To create a score for the number of chronic conditions, a summary score was calculated by summing the number of reported conditions. This measure included the 7 chronic conditions above and overweight/obesity (range 0-8).

*Physical functioning limitations.* Physical functioning limitations were assessed using items from scales developed by Rosow and Breslau (1966), Nagi (1976), Katz, Ford, Moskowitz, Jackson, and Jaffe (1963), and Lawton and Brody (1969).^3–6^ A total of 15 questions about physical functioning (e.g., walking several blocks, climbing one flight of stairs, pushing or pulling large objects, lifting or carrying 10 pounds, getting up from a chair, reaching or extending arms up, stooping, kneeling, or crouching, sitting for 2 hours) and activities of daily living (e.g., walking across a room, dressing, eating, bathing, getting in/out bed, using the toilet, picking up a dime) were included. Participants were classified as having “physical functioning limitations” if they reported >4 limitations with physical functioning, while participants who reported <4 limitations were considered “normal” (the reference group). This criterion was determined by identifying the physical function score where 75% of participants could be considered as having healthy physical function at baseline in the HRS sample.

*Cognitive impairment*. The HRS cognitive functioning assessment^7,8^ was adapted from the modified Telephone Interview for Cognitive Status (TICS-M). The assessment included an immediate and delayed 10-noun free recall test, a serial 7 subtraction test, and a backward count 20 test (27-point scale overall). This assessment tool has been shown to have high sensitivity and specificity when assessing cognitive impairment in older adults. The cut-off points used in this study were derived from previous research on cognitive impairment in HRS.^9,10^ Participants who scored 0-11 (on the 27-point scale) were classified as having “cognitive impairment,” while participants who scored ≥12 were classified as “normal” (the reference group). HRS reports contain further information about these cognitive assessments.^7,8^

*Chronic pain*. Participants were asked (yes/no), “Are you often troubled with pain?” The reference group was “no” pain.

*Self-rated health.* Participants were asked, “Would you say your health is excellent, very good, good, fair, or poor?” on a 5-point scale (reverse coded with higher scores indicating higher self-rated health).

*Hearing.* Participants were asked, “Is your hearing excellent, very good, good, fair, or poor (using a hearing aid as usual)?” on a 5-point Likert scale (reverse coded with higher values indicating better hearing). Self-report measures of hearing have been found to be reliable measures of hearing impairment.^11^

*Eyesight.* Participants were asked, “Is your eyesight excellent, very good, good, fair, or poor using glasses or corrective lenses as usual?” Response categories were as follows: 1) Excellent, 2) Very good, 3) Good, 4) Fair, 5) Poor or 6) Legally blind. Responses were reverse coded such that higher values were associated with better eyesight.

**Health Behaviors**

*Frequent physical activity*. Based on prior research, a binary physical activity variable was created: ≥1x/week of vigorous or moderate exercise was considered frequent physical activity, while <1x/week of vigorous or moderate exercise was the reference group.^12^ Participants indicated the frequency (i.e., response categories: daily, >1x/week, 1x/week, 1-3x/month, hardly ever or never) with which they engaged in vigorous (e.g., running, swimming, aerobics), moderate (e.g., gardening, dancing, walking at a moderate pace), and light (e.g., vacuuming, laundry) activities over the past 12 months.

*Smoking*. Participants were asked (yes/no), “Do you smoke cigarettes now?” to assess current smoking status. The reference group was “no” smoking.

*Heavy drinking.* Following the National Institute on Alcohol Abuse and Alcoholism guidelines,^13^ heavy drinking was defined as >14 for drinks/week for men and >7 drinks/week for women. Alcohol consumption was measured by multiplying the number of days/week that alcohol was consumed x number of drinks/day, which resulted in the number of drinks/week. Participants not in this alcohol consumption range were classified as non-heavy drinkers (the reference group).

*Sleep problems.* Participants completed the 4-item Jenkins Sleep Questionnaire, a widely used and validated screening instrument for assessing sleep complaints and insomnia symptoms.^14^ Response categories included “most of the time,” “sometimes,” and “rarely or never.” Having sleep problems was defined as reporting: “most of the time” for any of the three negatively worded items (e.g., “How often do you have trouble falling asleep?”) and “rarely or never” to the one positively worded item (i.e., “feel really rested when you wake up in the morning”). Participants were considered unhealthy (i.e., having sleep problems) if they reported one or more sleep problems. The sleep questionnaire was only administered every other wave. Thus, sleep data was imputed for half of the sample. Imputed and complete-case analyses showed similar estimates.

**Psychological Well-Being**

*Positive affect.* Positive affect was measured (in 2006 only) with a 6-item scale^15–17^ originally developed for use in the Midlife in the United States Study. The scale assessed how often the participant felt “cheerful,” “in good spirits,” “extremely happy,” “calm and peaceful,” “satisfied,” and “full of life” over the past 30 days. Response categories ranged from 1 (all of the time) to 5 (none of the time). Responses were reverse scored, so that a higher score indicated higher positive affect. An overall score was derived by averaging responses across all 6 items (α=0.91, range=1 to 5). After the 2006 wave, the HRS switched to a more expansive measure of positive affect based on the Positive and Negative Affect Schedule (PANAS-X).^18^ It included the following 13 items: determined, enthusiastic, active, proud, interested, happy, attentive, content, inspired, hopeful, alert, calm, and excited. An overall score was derived by averaging responses across all 13 items (α=0.92, range=1 to 5). A limitation of this study is that affect was measured in a different way during only the first wave of the study. However, scores were standardized and both the prior and current measures of affect operate very similarly (e.g., similar correlations with other variables, similar pattern of descriptive statistics).

*Life satisfaction.* Life satisfaction was assessed with the 5-item Satisfaction with Life Scale.^19^ The scale has shown excellent psychometric properties in prior work. Using a 7-point Likert scale (from 1 (strongly disagree) to 7 (strongly agree)), participants were asked the extent to which they agreed with statements such as, “In most ways my life is close to ideal.” Responses to all items were averaged to create a composite score, with higher scores indicating higher life satisfaction (α=0.88, range 1-7).

*Optimism.* Optimism was assessed with the Life Orientation Test-Revised (LOT-R), which has good discriminant and convergent validity, as well as good reliability.^20^ Using a 6-point Likert scale (from 1 (strongly disagree) to 6 (strongly agree)), participants were asked the degree to which they agreed with statements such as, “In uncertain times, I usually expect the best.” Negatively worded items were reverse coded and responses to all items were averaged to create an overall score, with higher scores indicating higher optimism (α=0.75, range 1-6).

*Purpose in life.* Purpose in life was assessed with a 7-item purpose in life subscale from Ryff’s Psychological Well-Being Scale.^21^ The 7-item subscale has been validated in prior work and has shown good psychometric properties.^22^ Using a 6-point Likert scale (from 1 (strongly disagree) to 6 (strongly agree)), participants were asked the degree to which they agreed with statements such as, “I have a sense of direction and purpose in my life.” Negatively worded items were reverse coded and all items were averaged to create a composite score, with higher scores indicating higher purpose (α=0.77, range 1-6).

*Mastery.* Mastery was assessed with 5-items derived from Lachman and Weaver (1998). The measure has good discriminant and convergent validity, and good reliability.^23^ Using a 6-point Likert scale (from 1 (strongly disagree) to 6 (strongly agree)), participants were asked the degree to which they agreed with statements such as, “I can do just about anything I really set my mind to.” All items were averaged to create a composite score, with higher scores indicating higher mastery (α=0.90, range 1-6).

*Health mastery.* Participants were asked, “How would you rate the amount of control you have over your health these days?” on a 0 (“no control at all”) to 10 (“very much control”) scale.

*Financial mastery.* Participants were asked, “How would you rate the amount of control you have over your financial situation these days?” on a 0 (“no control at all”) to 10 (“very much control”) scale.

**Psychological Distress**

*Depressive symptoms and depression.* Depressive symptoms were measured using The Center for Epidemiologic Studies Depression Scale (CESD).^24^ This scale has been validated in the HRS.^25^ Participants indicated the presence of 8 depressive symptoms (e.g., “Much of the time during the past week, I felt depressed”) over the past week (yes/no). All items were summed, with higher scores indicating higher depressive symptoms (α=0.80, range 0-8). Participants with scores of ≥4 were classified as having depression, as done previously (no depression was the reference group).^25^ Prior work has suggested that the cutoff value of 4 would produce results similar to the 16-item cutoff when using the full (20-item) CESD scale.^25^

*Hopelessness.* Hopelessness was assessed with a 4-item questionnaire from two previously validated scales.^26,27^ Using a 6-point Likert scale (from 1 (strongly disagree) to 6 (strongly agree)), participants were asked the degree to which they agree with statements such as, “The future seems hopeless to me and I can’t believe that things are changing for the better.” All items were averaged to create a composite score (α=0.86, range 1-6), with higher scores indicating more hopelessness.

*Negative affect*. Negative affect was measured (in 2006 only) with a 6-item scale originally developed for use in the Midlife in the United States Study.^15–17^ The scale assessed how often the participant felt “so depressed that nothing could cheer you up,” “hopeless,” “restless or fidgety,” “that everything was an effort,” “worthless,” and “nervous” over the past 30 days. Response categories ranged from 1 (all of the time) to 5 (none of the time). Responses were reverse scored, so that a higher score indicated higher negative affect. An overall score was derived by averaging responses across all 6 items (α=0.87, range=1 to 5). After the 2006 wave, the HRS switched to a more expansive measure of negative affect based on the Positive and Negative Affect Schedule (PANAS-X).^18^ It included the following 12 items: afraid, upset, guilty, scared, frustrated, bored, hostile, jittery, ashamed, nervous, sad, and distressed. An overall score was derived by averaging responses across all 12 items (α=0.89, range=1 to 5). A limitation of this study is that affect was measured in a different way during only the first wave of the study. However, scores were standardized and both the prior and current measures of affect operate very similarly (e.g., similar correlations with other variables, similar pattern of descriptive statistics).

*Perceived constraints.* Perceived constraints were assessed with 5 other items derived from Lachman and Weaver (1998), and this measure has good discriminant and convergent validity, as well as good reliability.^23^ Using a 6-point Likert scale (from 1 (strongly disagree) to 6 (strongly agree)), participants were asked the degree to which they agreed with statements such as, “What happens in my life is often beyond my control.” All items were averaged to create an overall score, with higher scores indicating a higher sense of constraints on personal control (α=0.87, range 1-6).

*Anxiety.* Anxiety was assessed using 5 of the 21 items in the Beck Anxiety Inventory (BAI).^28^ This inventory has been shown to differentiate between symptoms of depression and anxiety and has been validated in older adults.^29^ Participants were asked, “How often did you feel that way during the past week.” 1) “I had fear of the worst happening,” 2) “I was nervous,” 3) “I felt my hands trembling,” 4) “I had a fear of dying,” and 5) “I felt faint,” and could respond with 1 of 4 categories: 1) Never, 2) Hardly ever, 3) Some of the time, 4) Most of the time. The five responses were averaged, with higher scores indicating greater anxiety symptoms (α=0.81, range 1-4).

*Trait anger and state anger.* Trait anger (anger-in) and state anger (anger-out) are the two dimensions along which the Spielberger Anger Expression Scale (STAX) measures anger.^30^ These two dimensions have been shown to be separate factors that are modestly correlated through a principal factor analysis with Promax rotation.^31^ Trait anger is the predisposition to respond with anger across a variety of situations. To measure this variable, participants were asked to respond to four statements such as, “When I am feeling angry or mad, I keep things in.” State anger is a temporary behavioral reaction of anger and was measured through seven statements including, “When I am feeling angry or mad, I strike out at whatever infuriates me.” Participants gave responses on a 4-point Likert scale for each item: 1) Almost never, 2) Sometimes, 3) Often and 4) Almost always. Responses were averaged for trait anger (α=0.80) and state anger (α=0.82) separately, with higher scores indicating higher trait anger and state anger (range 1-4).

*Cynical hostility.* Cynical hostility was measured using 5 items from the Cook-Medley Hostility Inventory.^32^ The items were as follows: 1) “Most people dislike putting themselves out to help other people,” 2) “Most people will use somewhat unfair means to gain profit or an advantage rather than lose it,” 3) “No one cares much what happens to you,” 4) “I think most people would lie in order to get ahead,” and 5) “I commonly wonder what hidden reasons another person may have for doing something nice for me.” The first statement was written as, “Most people inwardly dislike putting themselves out to help other people” in the 2006 and 2008 questionnaire before being changed from 2010 onwards. Participants responded on a 6-point Likert scale (from 1 (strongly disagree) to 6 (strongly agree)). The scores were averaged (α=0.78, range 1-6), with higher scores indicating higher cynical hostility.

*Stressful life events.* Stressful life events were measured using 5 questions that have been used in other widely-used self-report measures of life stress.^33^ Items included questions such as, “Have you been unemployed and looking for work for longer than 3 months at some point in the past five years?” While the questionnaire in 2008 onwards asked an additional question of, “Have you been the victim of fraud in the past five years?”, this was not included for the purposes of the present study to maintain consistency (since it was not included in the 2006 questionnaire). Participants answered each question with a yes or no. Responses (0 = no, 1 = yes) were summed, with higher values indicating a higher number of stressful life events.

*Financial strain.* Respondents were asked, “How difficult is it for (you/your family) to meet monthly payments on (your/your family’s) bills?” and response options included: 1) Not at all difficult, 2) Not very difficult, 3) Somewhat difficult, 4) Very difficult or 5) Completely difficult. Higher scores indicated more financial strain.

*Daily discrimination and major discrimination.* Items measuring daily discrimination and major discrimination were based on prior widely used discrimination assessments.^34–36^ Daily discrimination was measured using 5 items that capture the frequency of the following experiences in the day-to-day lives of participants: 1) being treated with less courtesy or respect, 2) receiving poorer service in restaurants or stores, 3) people acting as if you are not smart, 4) people acting as if they are afraid of you, and 5) being threatened or harassed. Participants answered with one of the following response categories: 1) Almost every day, 2) At least once a week, 3) A few times a month, 4) A few times a year, 5) Less than once a year and 6) Never. Items were reverse-coded and averaged (α=0.80, range 1-6) such that higher scores indicated higher daily discrimination. The item, “You receive poorer service or treatment than other people from doctors or hospitals” (introduced in 2008) was excluded in the present study to maintain consistency as it was not present in the 2006 questionnaire. Major discrimination was measured using 6 items (yes/no) to capture major instances of lifetime discrimination: 1) being unfairly dismissed from a job, 2) not being hired for a job, 3) being unfairly denied a promotion, 4) being prevented from moving to a neighborhood because the realtor refused to sell/rent to you, 5) being unfairly denied a bank loan, and 6) being unfairly stopped by the police. Responses were summed with higher scores indicating more experiences of major discrimination. One item (“Have you ever been unfairly denied health care or treatment?” (introduced in 2008)) was excluded in the present study to maintain consistency as it was not included in the 2006 questionnaire.

**Social Factors**

*Living with a partner/spouse.* Participants were asked, “Do you have a husband, wife, or partner with whom you live?,” and answered yes/no.

*Frequency of contact with children/other family/friends.* The frequency of contact respondents had with members in their social network was evaluated through 3 items each for contacts who had 1) children, 2) other family, and 3) friends. Participants were asked, “On average, how often do you do each of the following?” 1) “Meet up (include both arranged and chance meetings),” 2) “Speak on the phone,” and 3) “Write or email.” Possible response categories were as follows: 1) Three or more times a week, 2) Once or twice a week, 3) Once or twice a month, 4) Every few months, 5) Once or twice a year or 6) Less than once a year or never. The responses were re-coded into the following categories: 0 = Every few months - never, 1 = 1-2x/month, 2 = 1-2x/week and 3 = 3 or more times/week.

*Loneliness.* Loneliness was assessed with three items from the previously validated UCLA Loneliness Scale.^37^ Participants were asked, “How much of the time do you feel”: 1) “you lack companionship”, 2) “left out”, and 3) “isolated from others”, with response categories ranging from 1 (often) to 3 (hardly ever or never). Responses were reverse scored and averaged, with higher scores indicating higher loneliness (α=0.80, range 1-3).

*Closeness with spouse.* One’s closeness with their spouse, if they had one, was assessed using a single question, “How close is your relationship with your spouse or partner?” Response options included: 1) Very close, 2) Quite close, 3) Not very close and 4) Not at all close. Responses were reverse coded to range from 1 (not at all close) to 4 (very close).

*Number of close children, close other family, close friends.* The quantity of close social ties was measured through the following 3 items: 1) “How many of your children would you say you have a close relationship with?”, 2) “How many of these family members would you say you have a close relationship with?”, and 3) “How many of your friends would you say you have a close relationship with?”

*Positive social support from spouse, children, other family, friends + Social strain from spouse, children, other family, friends.* The positive social support and negative social strain associated with each category of social ties were assessed using 3 and 4 items respectively. These items were based on those used in previous studies on social support.^38–40^ Items assessing positive social support were as follows: 1) “How much do they really understand the way you feel about things?”, 2) “How much can you rely on them if you have a serious problem?” and 3) “How much can you open up to them if you need to talk about your worries?” The 4 items assessing social strain were: 1) “How often do they make too many demands on you?”, 2) “How much do they criticize you?”, 3) “How much do they let you down when you are counting on them?” and 4) “How much do they get on your nerves?” Response options for all 7 questions included: 1) A lot, 2) Some, 3) A little or 4) Not at all. Scores were reverse coded and then averaged to create separate indexes for positive social support and negative social strain. Higher values indicated more positive social support or more social strain. This was done for positive social support from spouse (α=0.81), children (α=0.82), other family (α=0.82), and friends (α=0.84), as well as negative social strain from spouse (α=0.78), children (α=0.77), other family (α=0.78), and friends (α=0.75).

*Religious service attendance.* Participants were asked, “About how often have you attended religious services during the past year?” Possible response categories were as follows: 1) More than once a week, 2) Once a week, 3) Two or three times a month, 4) One or more times a year, or 5) Not at all. Response categories of 1 or 2 were redefined as “>or=1x/week.” Response categories of 3 or 4 were redefined as “<1x/week.” A response category of 5 was consistently defined as “Not at all.”

*Helping friends/neighbors/relatives.* Respondents were asked, “Have you spent any time in the past 12 months helping friends, neighbors, or relatives who did not live with you and did not pay you for the help?” If they answered yes to this question, respondents were asked how many hours they volunteered. Responses were coded as: 0 = 0 hours, 1 = 1-49 hours, 2 = 50-99 hours, 3 = 100-199 hours and 4 = ≥ 200 hours. Higher values indicated a greater amount of time spent helping others.

*Social status ladder + change in social status ladder.*  The MacArthur scale of subjective social status was used to evaluate an individual’s own position on the social ladder.^41^ Participants were asked to think of a ladder on which the people at the top were best off and those at the bottom were worst off based on money, education level and job quality (e.g., having one of the best jobs vs. having the worst jobs or no job). The first item asked respondents to place themselves on the ladder (range: 1-10). The second item asked, “Has your position on the ladder changed within the last two years?” Participants could answer 1) Yes, I have moved up, 2) Yes, I have moved down or 3) No, my position has not changed. Responses were re-coded into the following categories: 1 = downward movement, 2 = no change and 3 = upward movement.

**Work**

*In labor force.* Participants were asked, “Are you currently working?” An answer of 1 indicated “yes” while a 5 indicated “no”. Responses were recoded such that 1 = “In labor force” and 0 = “Not in labor force”.

**Supplementary Equations**

**Proof Illustrating How Adjusting for Pre-Baseline Levels of Exposure Can Help Us Evaluate How “Changes” in Exposure are Associated with Subsequent Volunteering Over Time**

Let Y be the volunteering outcome in 2014/2016, A_1_ the exposure under consideration in 2010/2012, A_0_ the prior level of exposure in 2006/2008, and C the set of all other covariates in 2006/2008.

For a binary outcome, the regression model is: log{P[Y=1|a_0_, a_1_, c]} = v + b_0_a_0_ + b_1_a_1_+ b_2_’c, which, if the outcome is rare can be approximated by a logistic regression model, logit{P[Y=1|a_0_, a_1_, c]} = v + b_0_a_0_ + b_1_a_1_+ b_2_’c, or, if the outcome is common, by a modified Poisson model. Let Y_a_ denote the potential outcome Y for an individual under an intervention to set A_1_ to a. For an individual with baseline exposure A_0_=a_0_ and covariates c in 2006/2008, under the no-confounding (and positivity and consistency) and modeling assumptions, a change in exposure of d points A_0_=a_0_ to A_1_=a_0_+d in 2010/2012, rather than maintaining exposure of A_1_=a_0_ in 2010/2012, will give rise to an effect on the risk ratio scale of:

P[Y_a0+d_=1| A_0_=a_0_, c] / P[Y_a0_=1| A_0_=a_0_, c]

= P[Y_a0+d_=1| A_1_=a_0_+d, A_0_=a_0_, c] / P[Y_a0_=1| A_1_=a_0_, A_0_=a_0_, c]

= P[Y=1| A_1_=a_0_+d, A_0_=a_0_, c] / P[Y=1| A_1_=a_0_, A_0_=a_0_, c]

= exp[v + b_0_a_0_ + b_1_(a_0_+d) + b_2_’c] / exp[v + b_0_a_0_ + b_1_a_0_ + b_2_’c]

= exp(b_1_d)

where the first equality follows by the no-confounding assumption, the second by consistency, and the third by the statistical model.

## Supplementary Information References

1. Fisher, G., Faul, J., Weir, D. & Wallace, R. Documentation of chronic disease measures in the Health and Retirement Study (HRS/AHEAD) (2005).

2. World Health Organization. Physical status: the use and interpretation of anthropometry: report of a WHO expert committee. *World Health Organ. Tech. Rep. Ser.* **854,** 1 (1995).

3. Rosow, I. & Breslau, N. A Guttman health scale for the aged. *J. Gerontol.* **21,** 556–559 (1966).

4. Nagi, S. An epidemiology of disability among adults in the United States. *Milbank Mem. Fund Q. Health Soc.* **54,** 439–467 (1976).

5. Katz, S., Ford, A. B., Moskowitz, R. W., Jackson, B. A. & Jaffe, M. W. Studies of illness in the aged: the index of ADL: a standardized measure of biological and psychosocial function. *JAMA* **185,** 914–919 (1963).

6. Lawton, M. P. & Brody, E. M. Assessment of older people: self-maintaining and instrumental activities of daily living. *Gerontologist* **9,** 179–186 (1969).

7. Fisher, G., Halimah, H., Faul, J., Rogers, W. & Weir, D. Health and Retirement Study imputation of cognitive functioning measures: 1992 – 2014 (2017).

8. Ofstedal, M., Fisher, G. & Herzog, A. Documentation of cognitive functioning measures in the Health and Retirement Study (2005).

9. Crimmins, E. M., Kim, J. K., Langa, K. M. & Weir, D. R. Assessment of cognition using surveys and neuropsychological assessment: the Health and Retirement Study and the Aging, Demographics, and Memory Study. *J. Gerontol. B. Psychol. Sci. Soc. Sci.* **66 Suppl 1,** i162-171 (2011).

10. Langa, K. M. *et al.* The Aging, Demographics, and Memory Study: study design and methods. *Neuroepidemiology* **25,** 181–191 (2005).

11. Chou, R., Dana, T., Bougatsos, C., Fleming, C. & Beil, T. Screening adults aged 50 years or older for hearing loss: a review of the evidence for the U.S. preventive services task force. *Ann. Intern. Med.* **154,** 347–355 (2011).

12. Nandi, A., Glymour, M. M. & Subramanian, S. V. Association among socioeconomic status, health behaviors, and all-cause mortality in the United States. *Epidemiol. Camb. Mass.* **25,** 170–177 (2014).

13. National Institute on Alcohol Abuse and Alcoholism (NIAAA). Drinking levels defined. https://www.niaaa.nih.gov/alcohol-health/overview-alcohol-consumption/moderate-binge-drinking.

14. Jenkins, C. D., Stanton, B. A., Niemcryk, S. J. & Rose, R. M. A scale for the estimation of sleep problems in clinical research. *J. Clin. Epidemiol.* **41,** 313–321 (1988).

15. Watson, D., Clark, L. A. & Tellegen, A. Development and validation of brief measures of positive and negative affect: the PANAS scales. *J. Pers. Soc. Psychol.* **54,** 1063–1070 (1988).

16. Brim, O. G. & Featherman, D. L. Surveying midlife development in the United States. *Unpubl. Manuscr.* (1998).

17. Mroczek, D. K. & Kolarz, C. M. The effect of age on positive and negative affect: a developmental perspective on happiness. *J. Pers. Soc. Psychol.* **75,** 1333–1349 (1998).

18. Watson, D. & Clark, L. A. The PANAS-X: Manual for the positive and negative affect schedule-expanded form (1994).

19. Diener, E., Emmons, R. A., Larsen, R. J. & Griffin, S. The Satisfaction With Life Scale. *J. Pers. Assess.* **49,** 71–75 (1985).

20. Scheier, M. F., Carver, C. S. & Bridges, M. W. Distinguishing optimism from neuroticism (and trait anxiety, self-mastery, and self-esteem): a reevaluation of the Life Orientation Test. *J. Pers. Soc. Psychol.* **67,** 1063–1078 (1994).

21. Ryff, C. D. & Keyes, C. L. M. The structure of psychological well-being revisited. *J. Pers. Soc. Psychol.* **69,** 719–727 (1995).

22. Abbott, R. *et al.* Psychometric evaluation and predictive validity of Ryff’s psychological well-being items in a UK birth cohort sample of women. *Health Qual. Life Outcomes* **4**, 76 (2006).

23. Lachman, M. E. & Weaver, S. L. The sense of control as a moderator of social class differences in health and well-being. *J. Pers. Soc. Psychol.* **74,** 763–773 (1998).

24. Radloff, L. S. The CES-D Scale: A self-report depression scale for research in the general population. *Appl. Psychol. Meas.* **1,** 385–401 (1977).

25. Steffeck, D. Documentation of affective functioning measures in the Health and Retirement Study (2000).

26. Beck, A. T., Weissman, A., Lester, D. & Trexler, L. The measurement of pessimism: the hopelessness scale. *J. Consult. Clin. Psychol.* **42,** 861–865 (1974).

27. Everson, S. A., Kaplan, G. A., Goldberg, D. E., Salonen, R. & Salonen, J. T. Hopelessness and 4-year progression of carotid atherosclerosis: the Kuopio Ischemic Heart Disease Risk Factor Study. *Arterioscler. Thromb. Vasc. Biol.* **17,** 1490–1495 (1997).

28. Beck, A. T., Epstein, N., Brown, G. & Steer, R. A. An inventory for measuring clinical anxiety: psychometric properties. *J. Consult. Clin. Psychol.* **56,** 893–897 (1988).

29. Wetherell, J. L. & Areán, P. A. Psychometric evaluation of the Beck Anxiety Inventory with older medical patients. *Psychol. Assess.* **9,** 136–144 (1997).

30. Forgays, D. K., Spielberger, C. D., Ottaway, S. A. & Forgays, D. G. Factor structure of the State-Trait Anger Expression Inventory for middle-aged men and women. *Assessment* **5,** 141–155 (1998).

31. Lee, Y. & Bierman, A. A longitudinal assessment of perceived discrimination and maladaptive expressions of anger among older adults: does subjective social power buffer the association? *J. Gerontol. Ser. B* **73,** e120–e130 (2018).

32. Cook, W. W. & Medley, D. M. Proposed hostility and Pharisaic-virtue scales for the MMPI. *J. Appl. Psychol.* **38,** 414–418 (1954).

33. Turner, R. J., Wheaton, B. & Lloyd, D. A. The epidemiology of social stress. *Am. Sociol. Rev.* **60,** 104–125 (1995).

34. Williams, D., Yan Y., Jackson, J. & Anderson, N. Racial differences in physical and mental health: socio-economic status, stress and discrimination. *J. Health Psychol.* **2,** 335–351 (1997).

35. Essed, P. *Understanding Everyday Racism: An Interdisciplinary Theory* (SAGE Publications, 1991).

36. Feagin, J. R. The continuing significance of race: antiblack discrimination in public places. *Am. Sociol. Rev.* **56,** 101–16 (1991).

37. Russell, D. W. UCLA Loneliness Scale (Version 3): reliability, validity, and factor structure. *J. Pers. Assess.* **66,** 20–40 (1996).

38. Schuster, T. L., Kessler, R. C. & Aseltine, R. H. Supportive interactions, negative interactions, and depressed mood. *Am. J. Community Psychol.* **18,** 423–438 (1990).

39. Smith, E. et al. Emotion theories and adolescent well-being: results of an online intervention. *Emotion (Washington D.C.)* **18,** 781-788 (2017).

40. Turner, R. J., Frankel, B. G. & Levin, D. M. Social support: conceptualization, measurement, and implications for mental health. *Res. Community Ment. Health* **3,** 67–111 (1983).

41. Adler, N. E. The MacArthur Scale of Subjective Social Status. https://macses.ucsf.edu/research/psychosocial/subjective.php.

**Supplementary Table 1. Changes in Volunteering from the Pre-Baseline Wave (t_0_) to the Outcome Wave (t_2_) – 4 Category Ordinal Volunteering Variable^a,b^**

|  | **Outcome Wave (t_2_)** | | | | |  |
| --- | --- | --- | --- | --- | --- | --- |
|  | | 0 h | 1-49 h | 50-99 h | >100 h | |
| **Pre-Baseline Wave (t_0_)** | |  |  |  |  | |
| 0 h | | 86.8 | 6.6 | 2.8 | 3.8 | |
| 1-49 h | | 55.7 | 19.9 | 11.5 | 13.0 | |
| 50-99 h | | 46.3 | 18.6 | 12.9 | 22.2 | |
| >100 h | | 31.8 | 11.9 | 13.2 | 43.1 | |

^a^The percent of people in category 1, 2, 3, or 4, in the pre-baseline wave (t_0_) who end up in category 1, 2, 3, or 4 in the outcome wave (t_2_).

^b^The values in the second (1-49 hours) row do not add up to 100% because of rounding.

**Supplementary Table 2. Changes in Candidate Predictors from the Pre-Baseline Wave (t_0_) to the Baseline Wave (t_1_)**

**Changes in Continuous Predictors from the Pre-Baseline Wave (t_0_) to the Baseline Wave (t_1_)**

| **Continuous Predictors** | **Pre-Baseline Wave (t_0_)** | **Baseline Wave (t_1_)** |
| --- | --- | --- |
|  | Mean (SD) | Mean (SD) |
| Number of physical conditions | 2.64 (1.5) | 3.02 (1.5) |
| Self-rated health | 3.15 (1.1) | 3.06 (1.1) |
| Hearing | 3.32 (1.1) | 3.20 (1.1) |
| Eyesight | 4.18 (1.0) | 4.09 (1.0) |
| Positive affect | 3.58 (0.7) | 3.48 (0.8) |
| Life satisfaction | 5.03 (1.5) | 4.83 (1.5) |
| Optimism | 4.46 (1.0) | 4.43 (1.0) |
| Purpose in life | 4.57 (0.9) | 4.49 (1.0) |
| Mastery | 4.75 (1.1) | 4.60 (1.2) |
| Health mastery | 7.23 (2.4) | 6.95 (2.5) |
| Financial mastery | 7.34 (2.7) | 7.21 (2.6) |
| Depressive symptoms | 1.41 (1.9) | 1.48 (1.9) |
| Hopelessness | 2.40 (1.3) | 2.47 (1.3) |
| Negative affect | 1.68 (0.6) | 1.74 (0.6) |
| Perceived constraints | 2.23 (1.2) | 2.32 (1.2) |
| Anxiety | 1.58 (0.6) | 1.59 (0.6) |
| Trait anger | 2.16 (0.7) | 2.14 (0.7) |
| State anger | 1.50 (0.5) | 1.47 (0.5) |
| Cynical hostility | 2.96 (1.1) | 2.89 (1.1) |
| Stressful life events | 0.23 (0.6) | 0.24 (0.6) |
| Financial strain | 1.96 (1.0) | 1.94 (1.0) |
| Daily discrimination | 1.63 (0.7) | 1.54 (0.7) |
| Major discrimination | 0.46 (0.9) | 0.39 (0.8) |
| Loneliness | 1.49 (0.5) | 1.49 (0.5) |
| Closeness with spouse | 3.25 (1.0) | 3.20 (1.0) |
| Number of close children | 2.75 (3.7) | 2.67 (3.2) |
| Number of close other family | 3.84 (5.6) | 3.80 (5.4) |
| Number of close friends | 4.37 (6.1) | 4.29 (5.7) |
| Positive social support from spouse | 3.27 (0.8) | 3.26 (0.8) |
| Positive social support from children | 3.22 (0.7) | 3.23 (0.7) |
| Positive social support from other family | 2.87 (0.9) | 2.87 (0.9) |
| Positive social support from friends | 3.01 (0.8) | 2.99 (0.8) |
| Social strain from spouse | 1.91 (0.7) | 1.86 (0.7) |
| Social strain from children | 1.71 (0.6) | 1.67 (0.6) |
| Social strain from other family | 1.57 (0.6) | 1.53 (0.6) |
| Social strain from friends | 1.83 (0.4) | 1.81 (0.4) |
| Social status ladder | 6.50 (1.8) | 6.39 (1.7) |

**Changes in Binary Predictors from the Pre-Baseline Wave (t_0_) to the Baseline Wave (t_1_)^a^**

|  | **Baseline Wave (t_1_)** | | | |
| --- | --- | --- | --- | --- |
|  | | No | Yes |  |
| **Pre-Baseline Wave (t_0_)** | |  |  |  |
| **Diabetes** | |  |  |  |
| No | | 93.3 | 6.7 |  |
| Yes | | 0.0 | 100.0 |  |
| **Hypertension** | |  |  |  |
| No | | 80.1 | 19.9 |  |
| Yes | | 0.1 | 99.9 |  |
| **Stroke** | |  |  |  |
| No | | 95.4 | 4.6 |  |
| Yes | | 0.0 | 100.0 |  |
| **Cancer** | |  |  |  |
| No | | 94.4 | 5.6 |  |
| Yes | | 0.0 | 100.0 |  |
| **Heart Disease** | |  |  |  |
| No | | 90.1 | 9.9 |  |
| Yes | | 0.0 | 100.0 |  |
| **Lung disease** | |  |  |  |
| No | | 96.1 | 3.9 |  |
| Yes | | 0.0 | 100.0 |  |
| **Arthritis** | |  |  |  |
| No | | 82.0 | 18.0 |  |
| Yes | | 0.1 | 99.9 |  |
| **Overweight/obese** | |  |  |  |
| No | | 84.3 | 15.7 |  |
| Yes | | 10.1 | 89.9 |  |
| **Physical functioning limitations** | |  |  |  |
| No | | 83.7 | 16.3 |  |
| Yes | | 21.4 | 78.7 |  |
| **Cognitive impairment** | |  |  |  |
| No | | 84.7 | 15.4 |  |
| Yes | | 28.0 | 72.0 |  |
| **Chronic pain** | |  |  |  |
| No | | 78.5 | 21.6 |  |
| Yes | | 31.1 | 68.9 |  |
| **Frequent physical activity** | |  |  |  |
| No | | 68.9 | 31.1 |  |
| Yes | | 25.5 | 74.5 |  |
| **Current smoking** | |  |  |  |
| No | | 99.0 | 1.0 |  |
| Yes | | 25.3 | 74.7 |  |
| **Heavy drinking** | |  |  |  |
| No | | 97.6 | 2.5 |  |
| Yes | | 45.8 | 54.2 |  |
| **Sleep problems** | |  |  |  |
| No | | 100.0 | 0.0 |  |
| Yes | | 0.0 | 100.0 |  |
| **Depression** | |  |  |  |
| No | | 91.6 | 8.4 |  |
| Yes | | 52.2 | 47.8 |  |
| **Living with a spouse/partner** | |  |  |  |
| No | | 94.7 | 5.3 |  |
| Yes | | 12.4 | 87.6 |  |
| **In Labor Force** | |  |  |  |
| No | | 96.3 | 3.7 |  |
| Yes | | 33.5 | 66.5 |  |

^a^Values in some rows for binary predictors do not add up to 100% because of rounding.

**Changes in Categorical Predictors from the Pre-Baseline Wave (t_0_) to the Baseline Wave (t_1_)^a^**

| **Contact children** | **Baseline Wave (t_1_)** | | | | |  |
| --- | --- | --- | --- | --- | --- | --- |
|  | | <Every few months | 1-2x/month | 1-2x/week | >3x/week | |
| **Pre-Baseline Wave (t_0_)** | |  |  |  |  | |
| <Every few months | | 74.4 | 10.0 | 8.9 | 6.7 | |
| 1-2x/month | | 19.3 | 34.0 | 33.2 | 13.5 | |
| 1-2x/week | | 5.9 | 13.3 | 48.8 | 32.0 | |
| >3x/week | | 2.6 | 4.2 | 21.9 | 71.3 | |

| **Contact other family** | **Baseline Wave (t_1_)** | | | | |  |
| --- | --- | --- | --- | --- | --- | --- |
|  | | <Every few months | 1-2x/month | 1-2x/week | >3x/week | |
| **Pre-Baseline Wave (t_0_)** | |  |  |  |  | |
| <Every few months | | 54.9 | 21.1 | 14.4 | 9.6 | |
| 1-2x/month | | 25.6 | 35.5 | 25.6 | 13.3 | |
| 1-2x/week | | 14.4 | 21.3 | 39.2 | 25.1 | |
| >3x/week | | 10.2 | 11.9 | 26.0 | 51.9 | |

| **Contact friends** | **Baseline Wave (t_1_)** | | | | |  |
| --- | --- | --- | --- | --- | --- | --- |
|  | | <Every few months | 1-2x/month | 1-2x/week | >3x/week | |
| **Pre-Baseline Wave (t_0_)** | |  |  |  |  | |
| <Every few months | | 53.6 | 19.9 | 18.1 | 8.4 | |
| 1-2x/month | | 22.3 | 32.1 | 32.3 | 13.4 | |
| 1-2x/week | | 10.7 | 18.8 | 44.1 | 26.4 | |
| >3x/week | | 5.7 | 8.3 | 30.9 | 55.1 | |

|  |  | **Baseline Wave (t_1_)** |  |
| --- | --- | --- | --- |
| **Religious service attendance** | Not at all | <1x/week | >1x/week |
| **Pre-Baseline Wave (t_0_)** |  |  |  |
| Not at all | 76.1 | 19.2 | 4.8 |
| <1x/week | 25.2 | 58.4 | 16.4 |
| >1x/week | 4.3 | 14.8 | 81.0 |

|  |  |  | **Baseline Wave (t_1_)** |  |  |
| --- | --- | --- | --- | --- | --- |
| **Helping friends/ neighbors/relatives** | 0 h/year | 1-49 h | 50-99 h | 100-199 h | >200 h |
| **Pre-Baseline Wave (t_0_)** |  |  |  |  |  |
| 0 h/year | 74.5 | 15.7 | 4.9 | 3.3 | 1.6 |
| 1-49 h/year | 40.2 | 38.4 | 12.7 | 5.6 | 3.1 |
| 50-99 h/year | 29.7 | 31.9 | 19.9 | 12.5 | 6.0 |
| 100-199 h/year | 27.8 | 26.8 | 19.8 | 16.4 | 9.3 |
| >200 h/year | 28.9 | 19.7 | 19.0 | 14.7 | 17.7 |

| **Change in social status ladder** |  | **Baseline Wave (t_1_)** |  |
| --- | --- | --- | --- |
|  | Yes, I have moved down | No, my position has not changed | Yes, I have moved up |
| **Pre-Baseline Wave (t_0_)** |  |  |  |
| Yes, I have moved down | 29.8 | 63.2 | 7.1 |
| No, my position has not changed | 10.9 | 82.3 | 6.8 |
| Yes, I have moved up | 12.2 | 68.4 | 19.4 |

^a^Values in some rows for categorical predictors do not add up to 100% because of rounding.

**Supplementary Table 3. Candidate Predictors of Volunteering: Among People Who Volunteer at Baseline (Health and Retirement Study [HRS]: N = 4,092)^a,b,c^**

| Candidate Predictor | Risk Ratio | 95% CI |
| --- | --- | --- |
| **Health Behaviors** |  |  |
| Frequent physical activity | 1.06 | 0.95, 1.18 |
| Smoking | 0.82 | 0.61, 1.11 |
| Heavy drinking | 1.08 | 0.88, 1.33 |
| Sleep problems | 0.99 | 0.91, 1.08 |
| **Physical Health** |  |  |
| Number of physical conditions | 1.00 | 0.91, 1.09 |
| Diabetes | 0.98 | 0.82, 1.17 |
| Hypertension | 1.02 | 0.88, 1.19 |
| Stroke | 0.86 | 0.64, 1.14 |
| Cancer | 0.98 | 0.81, 1.19 |
| Heart disease | 1.02 | 0.85, 1.22 |
| Lung disease | 0.91 | 0.66, 1.26 |
| Arthritis | 1.05 | 0.90, 1.22 |
| Overweight/obese | 1.01 | 0.88, 1.16 |
| Physical functioning limitations | 0.86 | 0.75, 0.99* |
| Cognitive impairment | 0.83 | 0.71, 0.96* |
| Chronic pain | 0.96 | 0.87, 1.06 |
| Self-rated health | 1.05 | 0.99, 1.12 |
| Hearing | 1.00 | 0.94, 1.06 |
| Eyesight | 1.01 | 0.96, 1.07 |
| **Psychological Well-being** |  |  |
| Positive affect | 1.08 | 1.02, 1.14* |
| Life satisfaction | 1.04 | 0.98, 1.10 |
| Optimism | 1.02 | 0.96, 1.08 |
| Purpose in life | 1.05 | 0.99, 1.13 |
| Mastery | 1.03 | 0.98, 1.09 |
| Health mastery | 1.02 | 0.96, 1.08 |
| Financial mastery | 1.02 | 0.97, 1.08 |
| **Psychological Distress** |  |  |
| Depression | 0.97 | 0.80, 1.16 |
| Depressive symptoms | 0.98 | 0.91, 1.04 |
| Hopelessness | 0.97 | 0.91, 1.03 |
| Negative affect | 0.97 | 0.91, 1.03 |
| Constraints | 0.94 | 0.89, 1.00* |
| Anxiety | 0.95 | 0.89, 1.01 |
| Trait anger | 0.97 | 0.93, 1.02 |
| State anger | 0.96 | 0.90, 1.03 |
| Cynical hostility | 0.99 | 0.94, 1.05 |
| Stressful life events | 1.00 | 0.96, 1.05 |
| Financial strain | 0.99 | 0.93, 1.06 |
| Daily discrimination | 0.99 | 0.93, 1.04 |
| Major discrimination | 1.01 | 0.96, 1.06 |
| **Social Factors** |  |  |
| Living with spouse/partner | 0.91 | 0.77, 1.06 |
| Contact children |  |  |
| <Every few months | Reference | Reference |
| 1-2x/month | 1.01 | 0.83, 1.23 |
| 1-2x/week | 1.06 | 0.89, 1.27 |
| >3x/week | 1.06 | 0.88, 1.27 |
| Contact other family |  |  |
| <Every few months | Reference | Reference |
| 1-2x/month | 1.04 | 0.92, 1.17 |
| 1-2x/week | 1.01 | 0.89, 1.15 |
| >3x/week | 1.01 | 0.88, 1.17 |
| Contact friends |  |  |
| <Every few months | Reference | Reference |
| 1-2x/month | 1.10 | 0.91, 1.32 |
| 1-2x/week | 1.15 | 0.97, 1.36 |
| >3x/week | 1.15 | 0.96, 1.37 |
| Loneliness | 0.98 | 0.93, 1.04 |
| Closeness with spouse | 0.98 | 0.91, 1.05 |
| Number of close children | 0.99 | 0.95, 1.04 |
| Number of close other family | 1.00 | 0.96, 1.04 |
| Number of close friends | 1.01 | 0.97, 1.05 |
| Positive social support from spouse | 1.01 | 0.95, 1.08 |
| Positive social support from children | 1.03 | 0.97, 1.09 |
| Positive social support from other family | 0.98 | 0.93, 1.03 |
| Positive social support from friends | 1.04 | 0.99, 1.10 |
| Social strain from spouse | 0.97 | 0.91, 1.04 |
| Social strain from children | 0.98 | 0.92, 1.04 |
| Social strain from other family | 1.00 | 0.95, 1.06 |
| Social strain from friends | 1.04 | 0.99, 1.09 |
| Religious service attendance |  |  |
| Not at all | Reference | Reference |
| <1x/week | 1.18 | 0.98, 1.43 |
| >1x/week | 1.41 | 1.16, 1.73*** |
| Helping friends/neighbors/relatives |  |  |
| 0 h/year | Reference | Reference |
| 1-49 h/year | 1.02 | 0.92, 1.14 |
| 50-99 h/year | 1.11 | 0.98, 1.25 |
| 100-199 h/year | 1.12 | 0.97, 1.29 |
| >200 h/year | 1.17 | 0.99, 1.37 |
| Social status ladder | 1.03 | 0.98, 1.09 |
| Change in social status ladder |  |  |
| Moved down | Reference | Reference |
| No change | 0.98 | 0.86, 1.11 |
| Moved up | 1.00 | 0.83, 1.20 |
| **Work** |  |  |
| In labor force | 0.94 | 0.84, 1.05 |

**P*<.05 before Bonferroni correction; ** *P*<.01 before Bonferroni correction; ****P*<.05 after Bonferroni correction (the *P* value cutoff for Bonferroni correction is *P* = .05/61 predictors = *P* <.00081968).

Abbreviations: CI, confidence interval; RR, risk ratio.

^~~a~~^The analytic sample was restricted to those who 1) had participated in the pre-baseline wave (2006 or 2008) and 2) reported volunteering at baseline. Multiple imputation was performed to impute missing data on the exposures, covariates, and outcome. Candidate antecedents were assessed, one at a time, in wave 2 (2010/2012), and the outcome (volunteering) was assessed in wave 3 (2014/2016). The following covariates were controlled for at wave 1 (2006/2008): sociodemographic characteristics (age, sex, race/ethnicity, marital status, income, total wealth, level of education, health insurance, geographic region), childhood abuse, personality factors (openness, conscientiousness, extraversion, agreeableness, neuroticism), and all of the predictor variables, including: health behaviors (physical activity, smoking, overweight/obese, heavy drinking, sleep problems), physical health (heart disease, cancer, stroke, arthritis, hypertension, diabetes, lung disease, chronic pain, hearing, eyesight, self-rated health, physical functioning limitations, cognitive impairment), social factors (live with spouse, frequency of contact with children, frequency of contact with other family, frequency of contact with friends, loneliness, closeness with spouse, number of close children, number of close other family, number of close friends, positive social support from spouse, positive social support from children, positive social support from friends, positive social support from other family, social strain from spouse, social strain from children, social strain from other family, social strain from friends, religious service attendance, volunteering, helping friends/neighbors/relatives, perceived social status, change in perceived social status), psychological well-being factors (life satisfaction, positive affect, optimism, purpose in life, health mastery, financial mastery, mastery), psychological distress (depressive symptoms, hopelessness, negative affect, constraints, anxiety, trait anger, state anger, daily discrimination, major discrimination, cynical hostility, stressful life events, financial strain), and work (in labor force).

^b^All continuous candidate antecedents were standardized (mean = 0; standard deviation = 1).

^c^An exposure-wide analytic approach was used, and a separate model for each exposure was run. Because volunteering was a binary outcome with a prevalence of ≥10%, we ran a generalized linear model with a log link and Poisson distribution to estimate a RR.

**Supplementary Table 4.** **Candidate Predictors of Volunteering: Among People Who Do Not Volunteer at Baseline (Health and Retirement Study [HRS]: N = 7,357)^a,b,c^**

| Candidate Predictor | Risk Ratio | 95% CI |
| --- | --- | --- |
| **Health Behaviors** |  |  |
| Frequent physical activity | 1.13 | 0.95, 1.35 |
| Smoking | 0.89 | 0.56, 1.41 |
| Heavy drinking | 0.99 | 0.66, 1.49 |
| Sleep problems | 0.88 | 0.66, 1.17 |
| **Physical Health** |  |  |
| Number of physical conditions | 0.99 | 0.83, 1.17 |
| Diabetes | 0.91 | 0.63, 1.31 |
| Hypertension | 1.10 | 0.83, 1.45 |
| Stroke | 0.68 | 0.39, 1.19 |
| Cancer | 1.14 | 0.72, 1.82 |
| Heart disease | 1.00 | 0.69, 1.46 |
| Lung disease | 0.92 | 0.57, 1.48 |
| Arthritis | 1.17 | 0.87, 1.57 |
| Overweight/obese | 0.90 | 0.70, 1.14 |
| Physical functioning limitations | 0.91 | 0.73, 1.14 |
| Cognitive impairment | 0.82 | 0.64, 1.05 |
| Chronic pain | 1.09 | 0.90, 1.32 |
| Self-rated health | 0.93 | 0.84, 1.04 |
| Hearing | 1.01 | 0.90, 1.14 |
| Eyesight | 1.02 | 0.93, 1.13 |
| **Psychological Well-being** |  |  |
| Positive affect | 1.09 | 0.97, 1.21 |
| Life satisfaction | 1.06 | 0.96, 1.18 |
| Optimism | 1.02 | 0.91, 1.13 |
| Purpose in life | 1.13 | 1.00, 1.27* |
| Mastery | 1.02 | 0.93, 1.12 |
| Health mastery | 0.99 | 0.88, 1.13 |
| Financial mastery | 0.99 | 0.88, 1.10 |
| **Psychological Distress** |  |  |
| Depression | 0.99 | 0.76, 1.30 |
| Depressive symptoms | 0.97 | 0.87, 1.08 |
| Hopelessness | 0.97 | 0.86, 1.09 |
| Negative affect | 1.02 | 0.91, 1.14 |
| Constraints | 0.92 | 0.82, 1.03 |
| Anxiety | 1.01 | 0.91, 1.11 |
| Trait anger | 1.02 | 0.92, 1.14 |
| State anger | 0.95 | 0.87, 1.05 |
| Cynical hostility | 0.97 | 0.89, 1.07 |
| Stressful life events | 1.08 | 1.00, 1.16 |
| Financial strain | 1.01 | 0.90, 1.14 |
| Daily discrimination | 1.02 | 0.92, 1.13 |
| Major discrimination | 1.11 | 1.01, 1.22* |
| **Social Factors** |  |  |
| Living with spouse/partner | 0.83 | 0.62, 1.11 |
| Contact children |  |  |
| <Every few months | Reference | Reference |
| 1-2x/month | 1.00 | 0.59, 1.70 |
| 1-2x/week | 1.38 | 0.88, 2.16 |
| >3x/week | 1.12 | 0.70, 1.77 |
| Contact other family |  |  |
| <Every few months | Reference | Reference |
| 1-2x/month | 0.90 | 0.68, 1.20 |
| 1-2x/week | 0.96 | 0.74, 1.24 |
| >3x/week | 1.09 | 0.84, 1.42 |
| Contact friends |  |  |
| <Every few months | Reference | Reference |
| 1-2x/month | 1.29 | 0.88, 1.90 |
| 1-2x/week | 1.33 | 1.00, 1.77* |
| >3x/week | 1.49 | 1.09, 2.04* |
| Loneliness | 1.04 | 0.94, 1.15 |
| Closeness with spouse | 0.98 | 0.79, 1.23 |
| Number of close children | 1.01 | 0.91, 1.12 |
| Number of close other family | 1.07 | 1.00, 1.15 |
| Number of close friends | 1.03 | 0.94, 1.13 |
| Positive social support from spouse | 1.06 | 0.90, 1.26 |
| Positive social support from children | 1.09 | 0.98, 1.21 |
| Positive social support from other family | 1.07 | 0.97, 1.17 |
| Positive social support from friends | 1.10 | 1.00, 1.21 |
| Social strain from spouse | 0.95 | 0.84, 1.06 |
| Social strain from children | 0.97 | 0.88, 1.09 |
| Social strain from other family | 1.07 | 0.97, 1.17 |
| Social strain from friends | 1.04 | 0.94, 1.15 |
| Religious service attendance |  |  |
| Not at all | Reference | Reference |
| <1x/week | 1.48 | 1.15, 1.90** |
| >1x/week | 2.22 | 1.65, 2.97*** |
| Helping friends/neighbors/relatives |  |  |
| 0 h/year | Reference | Reference |
| 1-49 h/year | 1.27 | 1.03, 1.57* |
| 50-99 h/year | 1.37 | 1.07, 1.77* |
| 100-199 h/year | 1.38 | 1.01, 1.89* |
| >200 h/year | 1.15 | 0.79, 1.68 |
| Social status ladder | 1.01 | 0.90, 1.14 |
| Change in social status ladder |  |  |
| Moved down | Reference | Reference |
| No change | 0.91 | 0.67, 1.25 |
| Moved up | 0.97 | 0.63, 1.50 |
| **Work** |  |  |
| In labor force | 0.97 | 0.78, 1.21 |

**P*<.05 before Bonferroni correction; ** *P*<.01 before Bonferroni correction; ****P*<.05 after Bonferroni correction (the *P* value cutoff for Bonferroni correction is *P* = .05/61 predictors = *P* <.00081968).

Abbreviations: CI, confidence interval; RR, risk ratio.

^~~a~~^The analytic sample was restricted to those who 1) had participated in the pre-baseline wave (2006 or 2008) and 2) reported no volunteering at baseline. Multiple imputation was performed to impute missing data on the exposures, covariates, and outcome. Candidate antecedents were assessed, one at a time, in wave 2 (2010/2012), and the outcome (volunteering) was assessed in wave 3 (2014/2016). The following covariates were controlled for at wave 1 (2006/2008): sociodemographic characteristics (age, sex, race/ethnicity, marital status, income, total wealth, level of education, health insurance, geographic region), childhood abuse, personality factors (openness, conscientiousness, extraversion, agreeableness, neuroticism), and all of the predictor variables, including: health behaviors (physical activity, smoking, overweight/obese, heavy drinking, sleep problems), physical health (heart disease, cancer, stroke, arthritis, hypertension, diabetes, lung disease, chronic pain, hearing, eyesight, self-rated health, physical functioning limitations, cognitive impairment), social factors (live with spouse, frequency of contact with children, frequency of contact with other family, frequency of contact with friends, loneliness, closeness with spouse, number of close children, number of close other family, number of close friends, positive social support from spouse, positive social support from children, positive social support from friends, positive social support from other family, social strain from spouse, social strain from children, social strain from other family, social strain from friends, religious service attendance, volunteering, helping friends/neighbors/relatives, perceived social status, change in perceived social status), psychological well-being factors (life satisfaction, positive affect, optimism, purpose in life, health mastery, financial mastery, mastery), psychological distress (depressive symptoms, hopelessness, negative affect, constraints, anxiety, trait anger, state anger, daily discrimination, major discrimination, cynical hostility, stressful life events, financial strain), and work (in labor force).

^b^All continuous candidate antecedents were standardized (mean = 0; standard deviation = 1).

^c^An exposure-wide analytic approach was used, and a separate model for each exposure was run. Because volunteering was a binary outcome with a prevalence of ≥10%, we ran a generalized linear model with a log link and Poisson distribution to estimate a RR.

**Supplementary Table 5.** **Candidate Predictors of Volunteering 100-Hour Threshold (Health and Retirement Study [HRS]: N = 13,771)^a,b,c^**

| Candidate Predictor | Risk Ratio | 95% CI |
| --- | --- | --- |
| **Health Behaviors** |  |  |
| Frequent physical activity | 1.40 | 1.21, 1.62*** |
| Smoking | 0.78 | 0.53, 1.16 |
| Heavy drinking | 1.37 | 1.02, 1.83* |
| Sleep problems | 0.96 | 0.83, 1.12 |
| **Physical Health** |  |  |
| Number of physical conditions | 1.03 | 0.92, 1.16 |
| Diabetes | 0.97 | 0.78, 1.21 |
| Hypertension | 1.17 | 0.96, 1.41 |
| Stroke | 0.97 | 0.64, 1.49 |
| Cancer | 0.90 | 0.69, 1.18 |
| Heart disease | 0.86 | 0.63, 1.17 |
| Lung disease | 0.88 | 0.55, 1.40 |
| Arthritis | 1.08 | 0.88, 1.32 |
| Overweight/obese | 1.12 | 0.94, 1.32 |
| Physical functioning limitations | 0.71 | 0.57, 0.89** |
| Cognitive impairment | 0.70 | 0.55, 0.88** |
| Chronic pain | 0.98 | 0.86, 1.11 |
| Self-rated health | 1.14 | 1.04, 1.25** |
| Hearing | 1.01 | 0.93, 1.09 |
| Eyesight | 1.07 | 1.00, 1.15 |
| **Psychological Well-being** |  |  |
| Positive affect | 1.15 | 1.07, 1.24*** |
| Life satisfaction | 1.08 | 0.99, 1.17 |
| Optimism | 1.00 | 0.93, 1.07 |
| Purpose in life | 1.15 | 1.07, 1.24*** |
| Mastery | 1.04 | 0.97, 1.11 |
| Health mastery | 1.01 | 0.94, 1.08 |
| Financial mastery | 0.99 | 0.92, 1.06 |
| **Psychological Distress** |  |  |
| Depression | 0.78 | 0.59, 1.03 |
| Depressive symptoms | 0.91 | 0.83, 0.99* |
| Hopelessness | 0.93 | 0.86, 1.01 |
| Negative affect | 0.94 | 0.85, 1.04 |
| Constraints | 0.89 | 0.81, 0.97* |
| Anxiety | 0.94 | 0.86, 1.03 |
| Trait anger | 0.99 | 0.93, 1.06 |
| State anger | 0.94 | 0.86, 1.04 |
| Cynical hostility | 0.94 | 0.86, 1.01 |
| Stressful life events | 0.99 | 0.94, 1.05 |
| Financial strain | 1.07 | 0.98, 1.16 |
| Daily discrimination | 1.00 | 0.93, 1.08 |
| Major discrimination | 1.01 | 0.95, 1.08 |
| **Social Factors** |  |  |
| Living with spouse/partner | 0.81 | 0.68, 0.97* |
| Contact children |  |  |
| <Every few months | Reference | Reference |
| 1-2x/month | 1.08 | 0.85, 1.38 |
| 1-2x/week | 1.16 | 0.93, 1.44 |
| >3x/week | 1.00 | 0.79, 1.27 |
| Contact other family |  |  |
| <Every few months | Reference | Reference |
| 1-2x/month | 1.10 | 0.93, 1.31 |
| 1-2x/week | 1.07 | 0.90, 1.27 |
| >3x/week | 1.02 | 0.83, 1.24 |
| Contact friends |  |  |
| <Every few months | Reference | Reference |
| 1-2x/month | 1.48 | 1.14, 1.94** |
| 1-2x/week | 1.76 | 1.34, 2.32*** |
| >3x/week | 1.94 | 1.45, 2.61*** |
| Loneliness | 0.99 | 0.92, 1.07 |
| Closeness with spouse | 0.94 | 0.81, 1.07 |
| Number of close children | 1.03 | 0.97, 1.09 |
| Number of close other family | 1.03 | 0.98, 1.09 |
| Number of close friends | 1.06 | 1.02, 1.11** |
| Positive social support from spouse | 1.00 | 0.92, 1.10 |
| Positive social support from children | 1.02 | 0.94, 1.10 |
| Positive social support from other family | 0.97 | 0.89, 1.04 |
| Positive social support from friends | 1.11 | 1.03, 1.20** |
| Social strain from spouse | 0.96 | 0.89, 1.04 |
| Social strain from children | 0.97 | 0.90, 1.05 |
| Social strain from other family | 1.02 | 0.95, 1.10 |
| Social strain from friends | 1.08 | 1.02, 1.15* |
| Religious service attendance |  |  |
| Not at all | Reference | Reference |
| <1x/week | 1.45 | 1.15, 1.82** |
| >1x/week | 2.51 | 1.81, 3.48*** |
| Helping friends/neighbors/relatives |  |  |
| 0 h/year | Reference | Reference |
| 1-49 h/year | 1.23 | 1.07, 1.41** |
| 50-99 h/year | 1.62 | 1.35, 1.94*** |
| 100-199 h/year | 1.82 | 1.52, 2.19*** |
| >200 h/year | 1.85 | 1.52, 2.26*** |
| Social status ladder | 1.01 | 0.94, 1.09 |
| Change in social status ladder |  |  |
| Moved down | Reference | Reference |
| No change | 0.95 | 0.79, 1.14 |
| Moved up | 0.82 | 0.64, 1.05 |
| **Work** |  |  |
| In labor force | 0.79 | 0.69, 0.92** |

**P*<.05 before Bonferroni correction; ** *P*<.01 before Bonferroni correction; ****P*<.05 after Bonferroni correction (the *P* value cutoff for Bonferroni correction is *P* = .05/61 predictors = *P* <.00081968).

Abbreviations: CI, confidence interval; RR, risk ratio.

^~~a~~^The analytic sample was restricted to those who had participated in the pre-baseline wave (2006 or 2008). Multiple imputation was performed to impute missing data on the exposures,

covariates, and outcome. Candidate antecedents were assessed, one at a time, in wave 2 (2010/2012), and the outcome (volunteering) was assessed in wave 3 (2014/2016). The following covariates were controlled for at wave 1 (2006/2008): sociodemographic characteristics (age, sex, race/ethnicity, marital status, income, total wealth, level of education, health insurance, geographic region), childhood abuse, personality factors (openness, conscientiousness, extraversion, agreeableness, neuroticism), and all of the predictor variables, including: health behaviors (physical activity, smoking, overweight/obese, heavy drinking, sleep problems), physical health (heart disease, cancer, stroke, arthritis, hypertension, diabetes, lung disease, chronic pain, hearing, eyesight, self-rated health, physical functioning limitations, cognitive impairment), social factors (live with spouse, frequency of contact with children, frequency of contact with other family, frequency of contact with friends, loneliness, closeness with spouse, number of close children, number of close other family, number of close friends, positive social support from spouse, positive social support from children, positive social support from friends, positive social support from other family, social strain from spouse, social strain from children, social strain from other family, social strain from friends, religious service attendance, volunteering, helping friends/neighbors/relatives, perceived social status, change in perceived social status), psychological well-being factors (life satisfaction, positive affect, optimism, purpose in life, health mastery, financial mastery, mastery), psychological distress (depressive symptoms, hopelessness, negative affect, constraints, anxiety, trait anger, state anger, daily discrimination, major discrimination, cynical hostility, stressful life events, financial strain), and work (in labor force).

^b^All continuous candidate antecedents were standardized (mean = 0; standard deviation = 1).

^c^An exposure-wide analytic approach was used, and a separate model for each exposure was run. Because volunteering was a binary outcome with a prevalence of ≥10%, we ran a generalized linear model with a log link and Poisson distribution to estimate a RR.

**Supplementary Table 6.** **Candidate Predictors of Volunteering: Among People Who Volunteer Regularly (>or=100 Hours/Week) at Baseline (Health and Retirement Study [HRS]: N = 1,679)^a,b,c^**

| Candidate Predictor | Risk Ratio | 95% CI |
| --- | --- | --- |
| **Health Behaviors** |  |  |
| Frequent physical activity | 1.05 | 0.88, 1.26 |
| Smoking | 0.85 | 0.51, 1.42 |
| Heavy drinking | 1.21 | 0.84, 1.75 |
| Sleep problems | 0.97 | 0.80, 1.18 |
| **Physical Health** |  |  |
| Number of physical conditions | 1.05 | 0.89, 1.23 |
| Diabetes | 1.01 | 0.74, 1.37 |
| Hypertension | 1.11 | 0.87, 1.42 |
| Stroke | 1.01 | 0.64, 1.61 |
| Cancer | 0.90 | 0.63, 1.29 |
| Heart disease | 1.12 | 0.79, 1.58 |
| Lung disease | 1.25 | 0.77, 2.03 |
| Arthritis | 1.05 | 0.81, 1.37 |
| Overweight/obese | 0.97 | 0.77, 1.22 |
| Physical functioning limitations | 0.85 | 0.66, 1.09 |
| Cognitive impairment | 0.82 | 0.61, 1.09 |
| Chronic pain | 0.97 | 0.82, 1.14 |
| Self-rated health | 1.04 | 0.93, 1.17 |
| Hearing | 0.99 | 0.90, 1.09 |
| Eyesight | 0.97 | 0.89, 1.06 |
| **Psychological Well-being** |  |  |
| Positive affect | 1.04 | 0.94, 1.16 |
| Life satisfaction | 1.03 | 0.93, 1.14 |
| Optimism | 1.00 | 0.91, 1.10 |
| Purpose in life | 1.07 | 0.96, 1.20 |
| Mastery | 1.01 | 0.92, 1.10 |
| Health mastery | 1.00 | 0.90, 1.11 |
| Financial mastery | 1.01 | 0.92, 1.12 |
| **Psychological Distress** |  |  |
| Depression | 1.08 | 0.75, 1.55 |
| Depressive symptoms | 0.97 | 0.86, 1.11 |
| Hopelessness | 0.97 | 0.86, 1.09 |
| Negative affect | 0.98 | 0.89, 1.09 |
| Constraints | 0.95 | 0.84, 1.07 |
| Anxiety | 1.02 | 0.91, 1.14 |
| Trait anger | 1.00 | 0.92, 1.09 |
| State anger | 0.94 | 0.83, 1.06 |
| Cynical hostility | 0.99 | 0.89, 1.09 |
| Stressful life events | 0.99 | 0.91, 1.08 |
| Financial strain | 1.05 | 0.95, 1.16 |
| Daily discrimination | 0.99 | 0.89, 1.09 |
| Major discrimination | 0.97 | 0.89, 1.06 |
| **Social Factors** |  |  |
| Living with spouse/partner | 0.86 | 0.68, 1.10 |
| Contact children |  |  |
| <Every few months | Reference | Reference |
| 1-2x/month | 1.05 | 0.74, 1.50 |
| 1-2x/week | 1.04 | 0.77, 1.40 |
| >3x/week | 1.00 | 0.72, 1.38 |
| Contact other family |  |  |
| <Every few months |  |  |
| 1-2x/month | 0.99 | 0.81, 1.20 |
| 1-2x/week | 1.05 | 0.86, 1.29 |
| >3x/week | 1.00 | 0.80, 1.26 |
| Contact friends |  |  |
| <Every few months | Reference | Reference |
| 1-2x/month | 1.04 | 0.73, 1.48 |
| 1-2x/week | 1.09 | 0.80, 1.50 |
| >3x/week | 1.15 | 0.82, 1.60 |
| Loneliness | 1.00 | 0.90, 1.11 |
| Closeness with spouse | 0.96 | 0.84, 1.10 |
| Number of close children | 1.04 | 0.96, 1.13 |
| Number of close other family | 1.02 | 0.96, 1.08 |
| Number of close friends | 1.04 | 0.97, 1.10 |
| Positive social support from spouse | 1.01 | 0.90, 1.12 |
| Positive social support from children | 1.04 | 0.95, 1.15 |
| Positive social support from other family | 0.96 | 0.88, 1.05 |
| Positive social support from friends | 1.04 | 0.94, 1.15 |
| Social strain from spouse | 0.98 | 0.89, 1.08 |
| Social strain from children | 0.95 | 0.87, 1.04 |
| Social strain from other family | 1.01 | 0.92, 1.11 |
| Social strain from friends | 1.06 | 0.97, 1.15 |
| Religious service attendance |  |  |
| Not at all | Reference | Reference |
| <1x/week | 1.16 | 0.85, 1.58 |
| >1x/week | 1.18 | 0.83, 1.67 |
| Helping friends/neighbors/relatives |  |  |
| 0 h/year | Reference | Reference |
| 1-49 h/year | 1.04 | 0.85, 1.28 |
| 50-99 h/year | 1.12 | 0.92, 1.37 |
| 100-199 h/year | 1.32 | 1.06, 1.65* |
| >200 h/year | 1.32 | 1.05, 1.67* |
| Social status ladder | 0.98 | 0.89, 1.08 |
| Change in social status ladder |  |  |
| Moved down | Reference | Reference |
| No change | 1.00 | 0.78, 1.29 |
| Moved up | 0.95 | 0.66, 1.36 |
| **Work** |  |  |
| In labor force | 0.89 | 0.73, 1.08 |

**P*<.05 before Bonferroni correction; ** *P*<.01 before Bonferroni correction; ****P*<.05 after Bonferroni correction (the *P* value cutoff for Bonferroni correction is *P* = .05/61 predictors = *P* <.00081968).

Abbreviations: CI, confidence interval; RR, risk ratio.

^~~a~~^The analytic sample was restricted to those who 1) had participated in the pre-baseline wave (2006 or 2008) and 2) reported volunteering regularly (>100 hours/week) at baseline. Multiple imputation was performed to impute missing data on the exposures, covariates, and outcome. Candidate antecedents were assessed, one at a time, in wave 2 (2010/2012), and the outcome (volunteering) was assessed in wave 3 (2014/2016). The following covariates were controlled for at wave 1 (2006/2008): sociodemographic characteristics (age, sex, race/ethnicity, marital status, income, total wealth, level of education, health insurance, geographic region), childhood abuse, personality factors (openness, conscientiousness, extraversion, agreeableness, neuroticism), and all of the predictor variables, including: health behaviors (physical activity, smoking, overweight/obese, heavy drinking, sleep problems), physical health (heart disease, cancer, stroke, arthritis, hypertension, diabetes, lung disease, chronic pain, hearing, eyesight, self-rated health, physical functioning limitations, cognitive impairment), social factors (live with spouse, frequency of contact with children, frequency of contact with other family, frequency of contact with friends, loneliness, closeness with spouse, number of close children, number of close other family, number of close friends, positive social support from spouse, positive social support from children, positive social support from friends, positive social support from other family, social strain from spouse, social strain from children, social strain from other family, social strain from friends, religious service attendance, volunteering, helping friends/neighbors/relatives, perceived social status, change in perceived social status), psychological well-being factors (life satisfaction, positive affect, optimism, purpose in life, health mastery, financial mastery, mastery), psychological distress (depressive symptoms, hopelessness, negative affect, constraints, anxiety, trait anger, state anger, daily discrimination, major discrimination, cynical hostility, stressful life events, financial strain), and work (in labor force).

^b^All continuous candidate antecedents were standardized (mean = 0; standard deviation = 1).

^c^An exposure-wide analytic approach was used, and a separate model for each exposure was run. Because volunteering was a binary outcome with a prevalence of ≥10%, we ran a generalized linear model with a log link and Poisson distribution to estimate a RR.

**Supplementary Table 7.** **Candidate Predictors of Volunteering: Among People Who Do Not Volunteer Regularly (<100 Hours/Week) at Baseline (Health and Retirement Study [HRS]: N = 9,770)^a,b,c^**

| Candidate Predictor | Odds Ratio | 95% CI |
| --- | --- | --- |
| **Health Behaviors** |  |  |
| Frequent physical activity | 1.68 | 1.26, 2.25*** |
| Smoking | 0.66 | 0.36, 1.21 |
| Heavy drinking | 1.85 | 1.14, 2.99* |
| Sleep problems | 0.94 | 0.67, 1.31 |
| **Physical Health** |  |  |
| Number of physical conditions | 1.10 | 0.88, 1.36 |
| Diabetes | 1.12 | 0.76, 1.67 |
| Hypertension | 1.24 | 0.86, 1.80 |
| Stroke | 1.08 | 0.53, 2.22 |
| Cancer | 1.00 | 0.64, 1.57 |
| Heart disease | 0.76 | 0.46, 1.25 |
| Lung disease | 0.50 | 0.18, 1.35 |
| Arthritis | 1.16 | 0.82, 1.65 |
| Overweight/obese | 1.25 | 0.91, 1.72 |
| Physical functioning limitations | 0.77 | 0.56, 1.06 |
| Cognitive impairment | 0.74 | 0.52, 1.04 |
| Chronic pain | 1.10 | 0.84, 1.44 |
| Self-rated health | 1.11 | 0.94, 1.31 |
| Hearing | 1.06 | 0.91, 1.24 |
| Eyesight | 1.20 | 1.06, 1.36** |
| **Psychological Well-being** |  |  |
| Positive affect | 1.23 | 1.06, 1.43** |
| Life satisfaction | 1.07 | 0.93, 1.23 |
| Optimism | 1.00 | 0.88, 1.14 |
| Purpose in life | 1.24 | 1.08, 1.44** |
| Mastery | 1.03 | 0.92, 1.16 |
| Health mastery | 0.95 | 0.83, 1.09 |
| Financial mastery | 0.90 | 0.80, 1.01 |
| **Psychological Distress** |  |  |
| Depression | 0.61 | 0.39, 0.94* |
| Depressive symptoms | 0.88 | 0.76, 1.01 |
| Hopelessness | 0.91 | 0.77, 1.08 |
| Negative affect | 0.89 | 0.73, 1.07 |
| Constraints | 0.86 | 0.75, 0.99* |
| Anxiety | 0.92 | 0.78, 1.09 |
| Trait anger | 1.00 | 0.87, 1.15 |
| State anger | 1.01 | 0.89, 1.15 |
| Cynical hostility | 0.96 | 0.84, 1.10 |
| Stressful life events | 1.02 | 0.92, 1.13 |
| Financial strain | 1.10 | 0.94, 1.28 |
| Daily discrimination | 1.06 | 0.93, 1.22 |
| Major discrimination | 1.06 | 0.93, 1.21 |
| **Social Factors** |  |  |
| Living with spouse/partner | 0.85 | 0.58, 1.23 |
| Contact children |  |  |
| <Every few months | Reference | Reference |
| 1-2x/month | 1.11 | 0.66, 1.88 |
| 1-2x/week | 1.57 | 1.00, 2.46* |
| >3x/week | 1.19 | 0.74, 1.91 |
| Contact other family |  |  |
| <Every few months | Reference | Reference |
| 1-2x/month | 1.34 | 1.01, 1.77* |
| 1-2x/week | 1.16 | 0.84, 1.62 |
| >3x/week | 1.12 | 0.78, 1.61 |
| Contact friends |  |  |
| <Every few months | Reference | Reference |
| 1-2x/month | 2.13 | 1.40, 3.23*** |
| 1-2x/week | 2.29 | 1.46, 3.58*** |
| >3x/week | 2.35 | 1.45, 3.81** |
| Loneliness | 0.99 | 0.87, 1.12 |
| Closeness with spouse | 0.92 | 0.70, 1.20 |
| Number of close children | 1.06 | 0.97, 1.17 |
| Number of close other family | 1.07 | 0.95, 1.19 |
| Number of close friends | 1.11 | 1.01, 1.22* |
| Positive social support from spouse | 1.00 | 0.84, 1.19 |
| Positive social support from children | 1.04 | 0.89, 1.21 |
| Positive social support from other family | 1.00 | 0.86, 1.17 |
| Positive social support from friends | 1.16 | 1.03, 1.31* |
| Social strain from spouse | 0.98 | 0.84, 1.13 |
| Social strain from children | 1.05 | 0.91, 1.20 |
| Social strain from other family | 1.02 | 0.90, 1.15 |
| Social strain from friends | 1.09 | 0.96, 1.24 |
| Religious service attendance |  |  |
| Not at all | Reference | Reference |
| <1x/week | 1.41 | 0.99, 2.02 |
| >1x/week | 3.55 | 2.18, 5.78*** |
| Helping friends/neighbors/relatives |  |  |
| 0 h/year | Reference | Reference |
| 1-49 h/year | 1.56 | 1.23, 1.98*** |
| 50-99 h/year | 1.80 | 1.27, 2.56*** |
| 100-199 h/year | 1.87 | 1.25, 2.81** |
| >200 h/year | 1.51 | 0.95, 2.42 |
| Social status ladder | 1.03 | 0.90, 1.19 |
| Change in social status ladder |  |  |
| Moved down | Reference | Reference |
| No change | 0.78 | 0.57, 1.06 |
| Moved up | 0.68 | 0.41, 1.13 |
| **Work** |  |  |
| In labor force | 0.76 | 0.59, 0.99* |

**P*<.05 before Bonferroni correction; ** *P*<.01 before Bonferroni correction; ****P*<.05 after Bonferroni correction (the *P* value cutoff for Bonferroni correction is *P* = .05/61 predictors = *P* <.00081968).

Abbreviations: CI, confidence interval; OR, odds ratio.

^~~a~~^The analytic sample was restricted to those who 1) had participated in the pre-baseline wave (2006 or 2008) and 2) reported <100 hours/year of volunteering at baseline. Multiple imputation was performed to impute missing data on the exposures, covariates, and outcome. Candidate antecedents were assessed, one at a time, in wave 2 (2010/2012), and the outcome (volunteering) was assessed in wave 3 (2014/2016). The following covariates were controlled for at wave 1 (2006/2008): sociodemographic characteristics (age, sex, race/ethnicity, marital status, income, total wealth, level of education, health insurance, geographic region), childhood abuse, personality factors (openness, conscientiousness, extraversion, agreeableness, neuroticism), and all of the predictor variables, including: health behaviors (physical activity, smoking, overweight/obese, heavy drinking, sleep problems), physical health (heart disease, cancer, stroke, arthritis, hypertension, diabetes, lung disease, chronic pain, hearing, eyesight, self-rated health, physical functioning limitations, cognitive impairment), social factors (live with spouse, frequency of contact with children, frequency of contact with other family, frequency of contact with friends, loneliness, closeness with spouse, number of close children, number of close other family, number of close friends, positive social support from spouse, positive social support from children, positive social support from friends, positive social support from other family, social strain from spouse, social strain from children, social strain from other family, social strain from friends, religious service attendance, volunteering, helping friends/neighbors/relatives, perceived social status, change in perceived social status), psychological well-being factors (life satisfaction, positive affect, optimism, purpose in life, health mastery, financial mastery, mastery), psychological distress (depressive symptoms, hopelessness, negative affect, constraints, anxiety, trait anger, state anger, daily discrimination, major discrimination, cynical hostility, stressful life events, financial strain), and work (in labor force).

^b^All continuous candidate antecedents were standardized (mean = 0; standard deviation = 1).

^c^An exposure-wide analytic approach was used, and a separate model for each exposure was run. Because volunteering was a binary outcome with a prevalence of <10%, we ran a logistic regression model to estimate an OR.

**Supplementary Table 8. Candidate Predictors of Volunteering – Ordinal Volunteering Variable (4 categories) (Health and Retirement Study [HRS]: N = 13,771)^a,b,c,d^**

| Candidate Predictor | Odds Ratio | 95% CI |
| --- | --- | --- |
| **Health Behaviors** |  |  |
| Frequent physical activity | 1.34 | 1.19, 1.51*** |
| Smoking | 0.71 | 0.53, 0.95* |
| Heavy drinking | 1.24 | 0.97, 1.58 |
| Sleep problems | 0.92 | 0.81, 1.06 |
| **Physical Health** |  |  |
| Number of physical conditions | 0.95 | 0.86, 1.05 |
| Diabetes | 0.85 | 0.69, 1.05 |
| Hypertension | 1.09 | 0.87, 1.37 |
| Stroke | 0.72 | 0.52, 1.00 |
| Cancer | 0.96 | 0.77, 1.20 |
| Heart disease | 0.91 | 0.71, 1.15 |
| Lung disease | 0.76 | 0.49, 1.17 |
| Arthritis | 1.07 | 0.88, 1.30 |
| Overweight/obese | 1.05 | 0.91, 1.21 |
| Physical functioning limitations | 0.67 | 0.58, 0.78*** |
| Cognitive impairment | 0.66 | 0.56, 0.77*** |
| Chronic pain | 0.93 | 0.79, 1.09 |
| Self-rated health | 1.16 | 1.09, 1.24*** |
| Hearing | 1.02 | 0.94, 1.10 |
| Eyesight | 1.09 | 1.02, 1.16* |
| **Psychological Well-being** |  |  |
| Positive affect | 1.25 | 1.17, 1.34*** |
| Life satisfaction | 1.14 | 1.05, 1.24** |
| Optimism | 1.05 | 0.98, 1.11 |
| Purpose in life | 1.22 | 1.13, 1.31*** |
| Mastery | 1.10 | 1.03, 1.16** |
| Health mastery | 1.06 | 0.99, 1.13 |
| Financial mastery | 1.04 | 0.98, 1.11 |
| **Psychological Distress** |  |  |
| Depression | 0.82 | 0.67, 1.01 |
| Depressive symptoms | 0.88 | 0.81, 0.96** |
| Hopelessness | 0.90 | 0.84, 0.96** |
| Negative affect | 0.92 | 0.83, 1.01 |
| Constraints | 0.83 | 0.75, 0.91*** |
| Anxiety | 0.89 | 0.83, 0.96** |
| Trait anger | 0.97 | 0.91, 1.03 |
| State anger | 0.90 | 0.83, 0.98* |
| Cynical hostility | 0.93 | 0.86, 1.01 |
| Stressful life events | 1.03 | 0.98, 1.08 |
| Financial strain | 1.03 | 0.94, 1.12 |
| Daily discrimination | 0.98 | 0.92, 1.04 |
| Major discrimination | 1.06 | 1.00, 1.12 |
| **Social Factors** |  |  |
| Living with spouse/partner | 0.80 | 0.65, 0.97* |
| Contact children |  |  |
| <Every few months | Reference | Reference |
| 1-2x/month | 1.10 | 0.85, 1.44 |
| 1-2x/week | 1.33 | 1.07, 1.66* |
| >3x/week | 1.15 | 0.93, 1.43 |
| Contact other family |  |  |
| <Every few months | Reference | Reference |
| 1-2x/month | 1.08 | 0.93, 1.26 |
| 1-2x/week | 1.07 | 0.90, 1.28 |
| >3x/week | 1.06 | 0.90, 1.24 |
| Contact friends |  |  |
| <Every few months | Reference | Reference |
| 1-2x/month | 1.48 | 1.25, 1.76*** |
| 1-2x/week | 1.77 | 1.51, 2.08*** |
| >3x/week | 2.01 | 1.69, 2.39*** |
| Loneliness | 0.98 | 0.91, 1.05 |
| Closeness with spouse | 0.95 | 0.77, 1.16 |
| Number of close children | 1.01 | 0.96, 1.07 |
| Number of close other family | 1.04 | 0.98, 1.10 |
| Number of close friends | 1.07 | 1.01, 1.13* |
| Positive social support from spouse | 1.08 | 0.98, 1.18 |
| Positive social support from children | 1.06 | 0.99, 1.13 |
| Positive social support from other family | 0.98 | 0.91, 1.05 |
| Positive social support from friends | 1.14 | 1.07, 1.21*** |
| Social strain from spouse | 0.93 | 0.84, 1.03 |
| Social strain from children | 0.94 | 0.88, 1.00* |
| Social strain from other family | 1.02 | 0.96, 1.09 |
| Social strain from friends | 1.10 | 1.03, 1.17** |
| Religious service attendance |  |  |
| Not at all | Reference | Reference |
| <1x/week | 1.68 | 1.39, 2.03*** |
| >1x/week | 3.73 | 3.05, 4.55*** |
| Helping friends/neighbors/relatives |  |  |
| 0 h/year | Reference | Reference |
| 1-49 h/year | 1.32 | 1.17, 1.47*** |
| 50-99 h/year | 1.77 | 1.50, 2.08*** |
| 100-199 h/year | 1.93 | 1.54, 2.41*** |
| >200 h/year | 2.03 | 1.62, 2.54*** |
| Social status ladder | 1.08 | 1.00, 1.16 |
| Change in social status ladder |  |  |
| Moved down | Reference | Reference |
| No change | 0.98 | 0.84, 1.14 |
| Moved up | 0.95 | 0.77, 1.17 |
| **Work** |  |  |
| In labor force | 0.83 | 0.73, 0.95** |

**P*<.05 before Bonferroni correction; ** *P*<.01 before Bonferroni correction; ****P*<.05 after Bonferroni correction (the *P* value cutoff for Bonferroni correction is

*P* = .05/61 predictors = *P* <.00081968).

Abbreviations: CI, confidence interval; OR, odds ratio.

^~~a~~^The analytic sample was restricted to those who had participated in the pre-baseline wave (2006 or 2008). Multiple imputation was performed to impute missing data on the exposures,

covariates, and outcome. Candidate antecedents were assessed, one at a time, in wave 2 (2010/2012), and the outcome (volunteering) was assessed in wave 3 (2014/2016). The following covariates were controlled for at wave 1 (2006/2008): sociodemographic characteristics (age, sex, race/ethnicity, marital status, income, total wealth, level of education, health insurance, geographic region), childhood abuse, personality factors (openness, conscientiousness, extraversion, agreeableness, neuroticism), and all of the predictor variables, including: health behaviors (physical activity, smoking, overweight/obese, heavy drinking, sleep problems), physical health (heart disease, cancer, stroke, arthritis, hypertension, diabetes, lung disease, chronic pain, hearing, eyesight, self-rated health, physical functioning limitations, cognitive impairment), social factors (live with spouse, frequency of contact with children, frequency of contact with other family, frequency of contact with friends, loneliness, closeness with spouse, number of close children, number of close other family, number of close friends, positive social support from spouse, positive social support from children, positive social support from friends, positive social support from other family, social strain from spouse, social strain from children, social strain from other family, social strain from friends, religious service attendance, volunteering, helping friends/neighbors/relatives, perceived social status, change in perceived social status), psychological well-being factors (life satisfaction, positive affect, optimism, purpose in life, health mastery, financial mastery, mastery), psychological distress (depressive symptoms, hopelessness, negative affect, constraints, anxiety, trait anger, state anger, daily discrimination, major discrimination, cynical hostility, stressful life events, financial strain), and work (in labor force).

^b^All continuous candidate antecedents were standardized (mean = 0; standard deviation = 1).

^c^An exposure-wide analytic approach was used, and a separate model for each exposure was run. Because volunteering was an ordinal outcome, we ran an ordinal logistic regression model to estimate an OR.

^d^These analyses were not included in the main paper as their results are more difficult to interpret (as these analyses represent movement between subcategories of volunteering).

**Supplementary Table 9. Candidate Predictors of Volunteering – Ordinal Volunteering Variable (4 categories): Among People Who Volunteer at Baseline (Health and Retirement Study [HRS]: N = 4,092)^a,b,c,d^**

| Candidate Predictor | Odds Ratio | 95% CI |
| --- | --- | --- |
| **Health Behaviors** |  |  |
| Frequent physical activity | 1.23 | 1.05, 1.44* |
| Smoking | 0.68 | 0.45, 1.05 |
| Heavy drinking | 1.35 | 0.95, 1.91 |
| Sleep problems | 0.98 | 0.82, 1.17 |
| **Physical Health** |  |  |
| Number of physical conditions | 1.03 | 0.89, 1.19 |
| Diabetes | 0.94 | 0.71, 1.25 |
| Hypertension | 1.11 | 0.88, 1.41 |
| Stroke | 0.81 | 0.49, 1.31 |
| Cancer | 0.86 | 0.63, 1.18 |
| Heart disease | 1.05 | 0.78, 1.43 |
| Lung disease | 0.90 | 0.56, 1.43 |
| Arthritis | 1.14 | 0.89, 1.46 |
| Overweight/obese | 1.09 | 0.88, 1.34 |
| Physical functioning limitations | 0.67 | 0.54, 0.82*** |
| Cognitive impairment | 0.63 | 0.51, 0.78*** |
| Chronic pain | 0.92 | 0.79, 1.08 |
| Self-rated health | 1.15 | 1.03, 1.29* |
| Hearing | 1.00 | 0.91, 1.11 |
| Eyesight | 1.02 | 0.94, 1.11 |
| **Psychological Well-being** |  |  |
| Positive affect | 1.25 | 1.12, 1.38*** |
| Life satisfaction | 1.11 | 1.03, 1.21* |
| Optimism | 1.03 | 0.94, 1.12 |
| Purpose in life | 1.20 | 1.08, 1.33** |
| Mastery | 1.09 | 1.01, 1.18* |
| Health mastery | 1.03 | 0.93, 1.13 |
| Financial mastery | 1.03 | 0.94, 1.14 |
| **Psychological Distress** |  |  |
| Depression | 0.91 | 0.68, 1.21 |
| Depressive symptoms | 0.93 | 0.85, 1.03 |
| Hopelessness | 0.92 | 0.83, 1.01 |
| Negative affect | 0.90 | 0.82, 0.98* |
| Constraints | 0.84 | 0.77, 0.92*** |
| Anxiety | 0.88 | 0.79, 0.97* |
| Trait anger | 0.96 | 0.88, 1.03 |
| State anger | 0.88 | 0.78, 0.99* |
| Cynical hostility | 0.97 | 0.88, 1.08 |
| Stressful life events | 0.99 | 0.93, 1.07 |
| Financial strain | 1.01 | 0.90, 1.15 |
| Daily discrimination | 0.98 | 0.90, 1.06 |
| Major discrimination | 1.01 | 0.92, 1.10 |
| **Social Factors** |  |  |
| Living with spouse/partner | 0.70 | 0.54, 0.90** |
| Contact children |  |  |
| <Every few months | Reference | Reference |
| 1-2x/month | 1.11 | 0.81, 1.51 |
| 1-2x/week | 1.27 | 0.97, 1.68 |
| >3x/week | 1.20 | 0.91, 1.60 |
| Contact other family |  |  |
| <Every few months | Reference | Reference |
| 1-2x/month | 1.12 | 0.91, 1.37 |
| 1-2x/week | 1.10 | 0.90, 1.35 |
| >3x/week | 1.05 | 0.82, 1.34 |
| Contact friends |  |  |
| <Every few months | Reference | Reference |
| 1-2x/month | 1.33 | 0.99, 1.80 |
| 1-2x/week | 1.67 | 1.28, 2.19*** |
| >3x/week | 1.74 | 1.30, 2.33*** |
| Loneliness | 0.97 | 0.89, 1.06 |
| Closeness with spouse | 0.92 | 0.75, 1.13 |
| Number of close children | 1.03 | 0.95, 1.12 |
| Number of close other family | 1.02 | 0.94, 1.10 |
| Number of close friends | 1.06 | 0.98, 1.13 |
| Positive social support from spouse | 1.03 | 0.90, 1.17 |
| Positive social support from children | 1.06 | 0.96, 1.17 |
| Positive social support from other family | 0.94 | 0.85, 1.03 |
| Positive social support from friends | 1.11 | 1.02, 1.21* |
| Social strain from spouse | 0.93 | 0.83, 1.05 |
| Social strain from children | 0.94 | 0.86, 1.03 |
| Social strain from other family | 1.02 | 0.93, 1.12 |
| Social strain from friends | 1.12 | 1.03, 1.21** |
| Religious service attendance |  |  |
| Not at all | Reference | Reference |
| <1x/week | 1.48 | 1.10, 1.99** |
| >1x/week | 2.46 | 1.83, 3.31*** |
| Helping friends/neighbors/relatives |  |  |
| 0 h/year | Reference | Reference |
| 1-49 h/year | 1.00 | 0.86, 1.17 |
| 50-99 h/year | 1.44 | 1.18, 1.74*** |
| 100-199 h/year | 1.90 | 1.49, 2.42*** |
| >200 h/year | 2.32 | 1.76, 3.06*** |
| Social status ladder | 1.06 | 0.97, 1.15 |
| Change in social status ladder |  |  |
| Moved down | Reference | Reference |
| No change | 0.94 | 0.77, 1.14 |
| Moved up | 0.87 | 0.60, 1.26 |
| **Work** |  |  |
| In labor force | 0.75 | 0.63, 0.89** |

**P*<.05 before Bonferroni correction; ** *P*<.01 before Bonferroni correction; ****P*<.05 after Bonferroni correction (the *P* value cutoff for Bonferroni correction is

*P* = .05/61 predictors = *P* <.00081968).

Abbreviations: CI, confidence interval; OR, odds ratio.

^~~a~~^The analytic sample was restricted to those who 1) had participated in the pre-baseline wave (2006 or 2008) and 2) reported volunteering at baseline. Multiple imputation was performed to impute missing data on the exposures, covariates, and outcome. Candidate antecedents were assessed, one at a time, in wave 2 (2010/2012), and the outcome (volunteering) was assessed in wave 3 (2014/2016). The following covariates were controlled for at wave 1 (2006/2008): sociodemographic characteristics (age, sex, race/ethnicity, marital status, income, total wealth, level of education, health insurance, geographic region), childhood abuse, personality factors (openness, conscientiousness, extraversion, agreeableness, neuroticism), and all of the predictor variables, including: health behaviors (physical activity, smoking, overweight/obese, heavy drinking, sleep problems), physical health (heart disease, cancer, stroke, arthritis, hypertension, diabetes, lung disease, chronic pain, hearing, eyesight, self-rated health, physical functioning limitations, cognitive impairment), social factors (live with spouse, frequency of contact with children, frequency of contact with other family, frequency of contact with friends, loneliness, closeness with spouse, number of close children, number of close other family, number of close friends, positive social support from spouse, positive social support from children, positive social support from friends, positive social support from other family, social strain from spouse, social strain from children, social strain from other family, social strain from friends, religious service attendance, volunteering, helping friends/neighbors/relatives, perceived social status, change in perceived social status), psychological well-being factors (life satisfaction, positive affect, optimism, purpose in life, health mastery, financial mastery, mastery), psychological distress (depressive symptoms, hopelessness, negative affect, constraints, anxiety, trait anger, state anger, daily discrimination, major discrimination, cynical hostility, stressful life events, financial strain), and work (in labor force).

^b^All continuous candidate antecedents were standardized (mean = 0; standard deviation = 1).

^c^An exposure-wide analytic approach was used, and a separate model for each exposure was run. Because volunteering was an ordinal outcome, we ran an ordinal logistic regression model to estimate an OR.

^d^These analyses were not included in the main paper as their results are more difficult to interpret (as these analyses represent movement between subcategories of volunteering).

**Supplementary Table 10. Candidate Predictors of Volunteering – Ordinal Volunteering Variable (4 categories): Among People Who Do Not Volunteer at Baseline (Health and Retirement Study [HRS]: N = 7,357)^a,b,c,d^**

| Candidate Predictor | Odds Ratio | 95% CI |
| --- | --- | --- |
| **Health Behaviors** |  |  |
| Frequent physical activity | 1.19 | 0.98, 1.44 |
| Smoking | 0.88 | 0.53, 1.46 |
| Heavy drinking | 1.09 | 0.68, 1.74 |
| Sleep problems | 0.86 | 0.62, 1.18 |
| **Physical Health** |  |  |
| Number of physical conditions | 0.99 | 0.81, 1.20 |
| Diabetes | 0.89 | 0.59, 1.35 |
| Hypertension | 1.16 | 0.84, 1.61 |
| Stroke | 0.64 | 0.35, 1.18 |
| Cancer | 1.16 | 0.66, 2.06 |
| Heart disease | 0.98 | 0.64, 1.48 |
| Lung disease | 0.85 | 0.49, 1.47 |
| Arthritis | 1.19 | 0.84, 1.69 |
| Overweight/obese | 0.90 | 0.68, 1.18 |
| Physical functioning limitations | 0.88 | 0.69, 1.12 |
| Cognitive impairment | 0.78 | 0.59, 1.03 |
| Chronic pain | 1.11 | 0.90, 1.39 |
| Self-rated health | 0.93 | 0.83, 1.05 |
| Hearing | 1.02 | 0.90, 1.16 |
| Eyesight | 1.05 | 0.94, 1.17 |
| **Psychological Well-being** |  |  |
| Positive affect | 1.13 | 1.00, 1.27 |
| Life satisfaction | 1.09 | 0.97, 1.22 |
| Optimism | 1.02 | 0.90, 1.16 |
| Purpose in life | 1.17 | 1.03, 1.34* |
| Mastery | 1.02 | 0.92, 1.14 |
| Health mastery | 0.99 | 0.86, 1.14 |
| Financial mastery | 0.98 | 0.87, 1.12 |
| **Psychological Distress** |  |  |
| Depression | 0.97 | 0.72, 1.31 |
| Depressive symptoms | 0.96 | 0.85, 1.08 |
| Hopelessness | 0.96 | 0.84, 1.09 |
| Negative affect | 1.02 | 0.89, 1.16 |
| Constraints | 0.89 | 0.79, 1.02 |
| Anxiety | 1.00 | 0.89, 1.11 |
| Trait anger | 1.02 | 0.90, 1.16 |
| State anger | 0.96 | 0.86, 1.06 |
| Cynical hostility | 0.96 | 0.86, 1.06 |
| Stressful life events | 1.10 | 1.00, 1.20* |
| Financial strain | 1.02 | 0.89, 1.17 |
| Daily discrimination | 1.02 | 0.91, 1.14 |
| Major discrimination | 1.14 | 1.01, 1.28* |
| **Social Factors** |  |  |
| Living with spouse/partner | 0.78 | 0.55, 1.09 |
| Contact children |  |  |
| <Every few months | Reference | Reference |
| 1-2x/month | 0.98 | 0.53, 1.83 |
| 1-2x/week | 1.49 | 0.87, 2.54 |
| >3x/week | 1.14 | 0.66, 1.97 |
| Contact other family |  |  |
| <Every few months | Reference | Reference |
| 1-2x/month | 0.90 | 0.64, 1.26 |
| 1-2x/week | 0.94 | 0.69, 1.28 |
| >3x/week | 1.11 | 0.83, 1.50 |
| Contact friends |  |  |
| <Every few months | Reference | Reference |
| 1-2x/month | 1.39 | 0.90, 2.17 |
| 1-2x/week | 1.44 | 1.05, 1.96* |
| >3x/week | 1.66 | 1.16, 2.37** |
| Loneliness | 1.04 | 0.93, 1.17 |
| Closeness with spouse | 0.98 | 0.75, 1.28 |
| Number of close children | 1.01 | 0.90, 1.14 |
| Number of close other family | 1.08 | 1.00, 1.18 |
| Number of close friends | 1.04 | 0.94, 1.15 |
| Positive social support from spouse | 1.08 | 0.89, 1.31 |
| Positive social support from children | 1.13 | 1.01, 1.26* |
| Positive social support from other family | 1.08 | 0.97, 1.21 |
| Positive social support from friends | 1.14 | 1.02, 1.26* |
| Social strain from spouse | 0.94 | 0.82, 1.07 |
| Social strain from children | 0.98 | 0.87, 1.11 |
| Social strain from other family | 1.08 | 0.98, 1.19 |
| Social strain from friends | 1.06 | 0.94, 1.20 |
| Religious service attendance |  |  |
| Not at all | Reference | Reference |
| <1x/week | 1.55 | 1.18, 2.04** |
| >1x/week | 2.61 | 1.87, 3.64*** |
| Helping friends/neighbors/relatives |  |  |
| 0 h/year | Reference | Reference |
| 1-49 h/year | 1.34 | 1.06, 1.69* |
| 50-99 h/year | 1.53 | 1.13, 2.08** |
| 100-199 h/year | 1.56 | 1.09, 2.22* |
| >200 h/year | 1.23 | 0.80, 1.89 |
| Social status ladder | 1.02 | 0.90, 1.17 |
| Change in social status ladder |  |  |
| Moved down | Reference | Reference |
| No change | 0.89 | 0.61, 1.31 |
| Moved up | 0.95 | 0.55, 1.63 |
| **Work** |  |  |
| In labor force | 0.97 | 0.75, 1.25 |

**P*<.05 before Bonferroni correction; ** *P*<.01 before Bonferroni correction; ****P*<.05 after Bonferroni correction (the *P* value cutoff for Bonferroni correction is

*P* = .05/61 predictors = *P* <.00081968).

Abbreviations: CI, confidence interval; OR, odds ratio.

^~~a~~^The analytic sample was restricted to those who 1) had participated in the pre-baseline wave (2006 or 2008) and 2) reported no volunteering at baseline. Multiple imputation was performed to impute missing data on the exposures, covariates, and outcome. Candidate antecedents were assessed, one at a time, in wave 2 (2010/2012), and the outcome (volunteering) was assessed in wave 3 (2014/2016). The following covariates were controlled for at wave 1 (2006/2008): sociodemographic characteristics (age, sex, race/ethnicity, marital status, income, total wealth, level of education, health insurance, geographic region), childhood abuse, personality factors (openness, conscientiousness, extraversion, agreeableness, neuroticism), and all of the predictor variables, including: health behaviors (physical activity, smoking, overweight/obese, heavy drinking, sleep problems), physical health (heart disease, cancer, stroke, arthritis, hypertension, diabetes, lung disease, chronic pain, hearing, eyesight, self-rated health, physical functioning limitations, cognitive impairment), social factors (live with spouse, frequency of contact with children, frequency of contact with other family, frequency of contact with friends, loneliness, closeness with spouse, number of close children, number of close other family, number of close friends, positive social support from spouse, positive social support from children, positive social support from friends, positive social support from other family, social strain from spouse, social strain from children, social strain from other family, social strain from friends, religious service attendance, volunteering, helping friends/neighbors/relatives, perceived social status, change in perceived social status), psychological well-being factors (life satisfaction, positive affect, optimism, purpose in life, health mastery, financial mastery, mastery), psychological distress (depressive symptoms, hopelessness, negative affect, constraints, anxiety, trait anger, state anger, daily discrimination, major discrimination, cynical hostility, stressful life events, financial strain), and work (in labor force).

^b^All continuous candidate antecedents were standardized (mean = 0; standard deviation = 1).

^c^An exposure-wide analytic approach was used, and a separate model for each exposure was run. Because volunteering was an ordinal outcome, we ran an ordinal logistic regression model to estimate an OR.

^d^These analyses were not included in the main paper as their results are more difficult to interpret (as these analyses represent movement between subcategories of volunteering).

**Supplementary Table 11. Candidate Predictors of Volunteering – Ordinal Volunteering Variable (3 categories) (Health and Retirement Study [HRS]: N = 13,771)^a,b,c,d^**

| Candidate Predictor | Odds Ratio | 95% CI |
| --- | --- | --- |
| **Health Behaviors** |  |  |
| Frequent physical activity | 1.35 | 1.19, 1.52*** |
| Smoking | 0.70 | 0.52, 0.94* |
| Heavy drinking | 1.25 | 0.98, 1.59 |
| Sleep problems | 0.91 | 0.80, 1.04 |
| **Physical Health** |  |  |
| Number of physical conditions | 0.95 | 0.85, 1.05 |
| Diabetes | 0.86 | 0.70, 1.05 |
| Hypertension | 1.09 | 0.86, 1.37 |
| Stroke | 0.71 | 0.51, 1.00* |
| Cancer | 0.96 | 0.76, 1.20 |
| Heart disease | 0.90 | 0.70, 1.16 |
| Lung disease | 0.77 | 0.49, 1.19 |
| Arthritis | 1.08 | 0.89, 1.32 |
| Overweight/obese | 1.05 | 0.90, 1.21 |
| Physical functioning limitations | 0.67 | 0.58, 0.78*** |
| Cognitive impairment | 0.67 | 0.57, 0.79*** |
| Chronic pain | 0.94 | 0.79, 1.11 |
| Self-rated health | 1.16 | 1.09, 1.25*** |
| Hearing | 1.02 | 0.94, 1.10 |
| Eyesight | 1.09 | 1.02, 1.16** |
| **Psychological Well-being** |  |  |
| Positive affect | 1.25 | 1.17, 1.34*** |
| Life satisfaction | 1.14 | 1.05, 1.24** |
| Optimism | 1.05 | 0.98, 1.11 |
| Purpose in life | 1.21 | 1.12, 1.30*** |
| Mastery | 1.10 | 1.03, 1.16** |
| Health mastery | 1.06 | 0.99, 1.13 |
| Financial mastery | 1.04 | 0.98, 1.11 |
| **Psychological Distress** |  |  |
| Depression | 0.82 | 0.67, 1.01 |
| Depressive symptoms | 0.88 | 0.81, 0.96** |
| Hopelessness | 0.90 | 0.84, 0.96** |
| Negative affect | 0.92 | 0.84, 1.01 |
| Constraints | 0.83 | 0.76, 0.91*** |
| Anxiety | 0.89 | 0.83, 0.96** |
| Trait anger | 0.97 | 0.91, 1.03 |
| State anger | 0.90 | 0.83, 0.98* |
| Cynical hostility | 0.93 | 0.86, 1.00* |
| Stressful life events | 1.03 | 0.98, 1.08 |
| Financial strain | 1.03 | 0.94, 1.12 |
| Daily discrimination | 0.98 | 0.92, 1.04 |
| Major discrimination | 1.06 | 1.00, 1.13* |
| **Social Factors** |  |  |
| Living with spouse/partner | 0.80 | 0.65, 0.97* |
| Contact children |  |  |
| <Every few months | Reference | Reference |
| 1-2x/month | 1.10 | 0.85, 1.42 |
| 1-2x/week | 1.31 | 1.06, 1.63* |
| >3x/week | 1.13 | 0.91, 1.40 |
| Contact other family |  |  |
| <Every few months | Reference | Reference |
| 1-2x/month | 1.08 | 0.93, 1.26 |
| 1-2x/week | 1.07 | 0.91, 1.26 |
| >3x/week | 1.05 | 0.90, 1.24 |
| Contact friends |  |  |
| <Every few months | Reference | Reference |
| 1-2x/month | 1.47 | 1.23, 1.74*** |
| 1-2x/week | 1.75 | 1.49, 2.05*** |
| >3x/week | 1.98 | 1.66, 2.36*** |
| Loneliness | 0.98 | 0.91, 1.05 |
| Closeness with spouse | 0.95 | 0.77, 1.16 |
| Number of close children | 1.02 | 0.96, 1.08 |
| Number of close other family | 1.04 | 0.98, 1.10 |
| Number of close friends | 1.07 | 1.02, 1.13* |
| Positive social support from spouse | 1.07 | 0.98, 1.18 |
| Positive social support from children | 1.06 | 0.99, 1.14 |
| Positive social support from other family | 0.97 | 0.91, 1.04 |
| Positive social support from friends | 1.14 | 1.07, 1.22*** |
| Social strain from spouse | 0.93 | 0.84, 1.02 |
| Social strain from children | 0.93 | 0.87, 1.00* |
| Social strain from other family | 1.02 | 0.96, 1.09 |
| Social strain from friends | 1.10 | 1.03, 1.17** |
| Religious service attendance |  |  |
| Not at all | Reference | Reference |
| <1x/week | 1.66 | 1.37, 2.01*** |
| >1x/week | 3.74 | 3.05, 4.58*** |
| Helping friends/neighbors/relatives |  |  |
| 0 h/year | Reference | Reference |
| 1-49 h/year | 1.33 | 1.19, 1.49*** |
| 50-99 h/year | 1.78 | 1.51, 2.09*** |
| 100-199 h/year | 1.93 | 1.53, 2.42*** |
| >200 h/year | 2.02 | 1.60, 2.54*** |
| Social status ladder | 1.08 | 0.99, 1.16 |
| Change in social status ladder |  |  |
| Moved down | Reference | Reference |
| No change | 0.99 | 0.84, 1.15 |
| Moved up | 0.96 | 0.77, 1.19 |
| **Work** |  |  |
| In labor force | 0.83 | 0.73, 0.94** |

**P*<.05 before Bonferroni correction; ** *P*<.01 before Bonferroni correction; ****P*<.05 after Bonferroni correction (the *P* value cutoff for Bonferroni correction is

*P* = .05/61 predictors = *P* <.00081968).

Abbreviations: CI, confidence interval; OR, odds ratio.

^~~a~~^The analytic sample was restricted to those who had participated in the pre-baseline wave (2006 or 2008). Multiple imputation was performed to impute missing data on the exposures,

covariates, and outcome. Candidate antecedents were assessed, one at a time, in wave 2 (2010/2012), and the outcome (volunteering) was assessed in wave 3 (2014/2016). The following covariates were controlled for at wave 1 (2006/2008): sociodemographic characteristics (age, sex, race/ethnicity, marital status, income, total wealth, level of education, health insurance, geographic region), childhood abuse, personality factors (openness, conscientiousness, extraversion, agreeableness, neuroticism), and all of the predictor variables, including: health behaviors (physical activity, smoking, overweight/obese, heavy drinking, sleep problems), physical health (heart disease, cancer, stroke, arthritis, hypertension, diabetes, lung disease, chronic pain, hearing, eyesight, self-rated health, physical functioning limitations, cognitive impairment), social factors (live with spouse, frequency of contact with children, frequency of contact with other family, frequency of contact with friends, loneliness, closeness with spouse, number of close children, number of close other family, number of close friends, positive social support from spouse, positive social support from children, positive social support from friends, positive social support from other family, social strain from spouse, social strain from children, social strain from other family, social strain from friends, religious service attendance, volunteering, helping friends/neighbors/relatives, perceived social status, change in perceived social status), psychological well-being factors (life satisfaction, positive affect, optimism, purpose in life, health mastery, financial mastery, mastery), psychological distress (depressive symptoms, hopelessness, negative affect, constraints, anxiety, trait anger, state anger, daily discrimination, major discrimination, cynical hostility, stressful life events, financial strain), and work (in labor force).

^b^All continuous candidate antecedents were standardized (mean = 0; standard deviation = 1).

^c^An exposure-wide analytic approach was used, and a separate model for each exposure was run. Because volunteering was an ordinal outcome, we ran an ordinal logistic regression model to estimate an OR.

^d^These analyses were not included in the main paper as their results are more difficult to interpret (as these analyses represent movement between subcategories of volunteering).

**Supplementary Table 12. Candidate Predictors of Volunteering – Ordinal Volunteering Variable (3 categories): Among People Who Volunteer at Baseline (Health and Retirement Study [HRS]: N = 4,092)^a,b,c,d^**

| Candidate Predictor | Odds Ratio | 95% CI |
| --- | --- | --- |
| **Health Behaviors** |  |  |
| Frequent physical activity | 1.24 | 1.05, 1.46** |
| Smoking | 0.67 | 0.43, 1.03 |
| Heavy drinking | 1.38 | 0.97, 1.94 |
| Sleep problems | 0.97 | 0.81, 1.15 |
| **Physical Health** |  |  |
| Number of physical conditions | 1.04 | 0.90, 1.20 |
| Diabetes | 0.98 | 0.74, 1.30 |
| Hypertension | 1.10 | 0.86, 1.40 |
| Stroke | 0.80 | 0.49, 1.32 |
| Cancer | 0.84 | 0.60, 1.16 |
| Heart disease | 1.05 | 0.77, 1.44 |
| Lung disease | 0.91 | 0.57, 1.46 |
| Arthritis | 1.17 | 0.91, 1.50 |
| Overweight/obese | 1.10 | 0.89, 1.36 |
| Physical functioning limitations | 0.67 | 0.55, 0.83*** |
| Cognitive impairment | 0.65 | 0.52, 0.81*** |
| Chronic pain | 0.93 | 0.79, 1.10 |
| Self-rated health | 1.15 | 1.03, 1.29* |
| Hearing | 1.00 | 0.90, 1.11 |
| Eyesight | 1.02 | 0.94, 1.11 |
| **Psychological Well-being** |  |  |
| Positive affect | 1.24 | 1.12, 1.38*** |
| Life satisfaction | 1.11 | 1.02, 1.21* |
| Optimism | 1.03 | 0.94, 1.13 |
| Purpose in life | 1.18 | 1.06, 1.31** |
| Mastery | 1.09 | 1.00, 1.18 |
| Health mastery | 1.02 | 0.92, 1.13 |
| Financial mastery | 1.03 | 0.94, 1.14 |
| **Psychological Distress** |  |  |
| Depression | 0.89 | 0.67, 1.19 |
| Depressive symptoms | 0.94 | 0.85, 1.04 |
| Hopelessness | 0.92 | 0.83, 1.02 |
| Negative affect | 0.91 | 0.82, 1.00* |
| Constraints | 0.85 | 0.78, 0.94** |
| Anxiety | 0.88 | 0.79, 0.97* |
| Trait anger | 0.96 | 0.88, 1.04 |
| State anger | 0.88 | 0.78, 0.99* |
| Cynical hostility | 0.96 | 0.87, 1.06 |
| Stressful life events | 1.00 | 0.93, 1.07 |
| Financial strain | 1.02 | 0.90, 1.16 |
| Daily discrimination | 0.97 | 0.89, 1.06 |
| Major discrimination | 1.01 | 0.92, 1.10 |
| **Social Factors** |  |  |
| Living with spouse/partner | 0.68 | 0.53, 0.88** |
| Contact children |  |  |
| <Every few months | Reference | Reference |
| 1-2x/month | 1.08 | 0.78, 1.48 |
| 1-2x/week | 1.22 | 0.93, 1.61 |
| >3x/week | 1.14 | 0.86, 1.52 |
| Contact other family |  |  |
| <Every few months | Reference | Reference |
| 1-2x/month | 1.11 | 0.90, 1.37 |
| 1-2x/week | 1.09 | 0.88, 1.35 |
| >3x/week | 1.04 | 0.81, 1.34 |
| Contact friends |  |  |
| <Every few months | Reference | Reference |
| 1-2x/month | 1.29 | 0.96, 1.73 |
| 1-2x/week | 1.60 | 1.23, 2.08*** |
| >3x/week | 1.65 | 1.24, 2.21** |
| Loneliness | 0.98 | 0.89, 1.07 |
| Closeness with spouse | 0.91 | 0.74, 1.13 |
| Number of close children | 1.04 | 0.96, 1.13 |
| Number of close other family | 1.02 | 0.94, 1.10 |
| Number of close friends | 1.06 | 0.99, 1.13 |
| Positive social support from spouse | 1.01 | 0.90, 1.15 |
| Positive social support from children | 1.07 | 0.97, 1.18 |
| Positive social support from other family | 0.93 | 0.85, 1.03 |
| Positive social support from friends | 1.12 | 1.03, 1.23* |
| Social strain from spouse | 0.93 | 0.83, 1.05 |
| Social strain from children | 0.93 | 0.85, 1.02 |
| Social strain from other family | 1.03 | 0.93, 1.13 |
| Social strain from friends | 1.12 | 1.03, 1.22** |
| Religious service attendance |  |  |
| Not at all | Reference | Reference |
| <1x/week | 1.43 | 1.06, 1.93* |
| >1x/week | 2.44 | 1.81, 3.28*** |
| Helping friends/neighbors/relatives |  |  |
| 0 h/year | Reference | Reference |
| 1-49 h/year | 1.03 | 0.88, 1.21 |
| 50-99 h/year | 1.45 | 1.19, 1.77*** |
| 100-199 h/year | 1.90 | 1.49, 2.42*** |
| >200 h/year | 2.33 | 1.76, 3.10*** |
| Social status ladder | 1.06 | 0.96, 1.15 |
| Change in social status ladder |  |  |
| Moved down | Reference | Reference |
| No change | 0.94 | 0.76, 1.16 |
| Moved up | 0.88 | 0.59, 1.32 |
| **Work** |  |  |
| In labor force | 0.74 | 0.62, 0.89** |

**P*<.05 before Bonferroni correction; ** *P*<.01 before Bonferroni correction; ****P*<.05 after Bonferroni correction (the *P* value cutoff for Bonferroni correction is

*P* = .05/61 predictors = *P* <.00081968).

Abbreviations: CI, confidence interval; OR, odds ratio.

^~~a~~^The analytic sample was restricted to those who 1) had participated in the pre-baseline wave (2006 or 2008) and 2) reported volunteering at baseline. Multiple imputation was performed to impute missing data on the exposures, covariates, and outcome. Candidate antecedents were assessed, one at a time, in wave 2 (2010/2012), and the outcome (volunteering) was assessed in wave 3 (2014/2016). The following covariates were controlled for at wave 1 (2006/2008): sociodemographic characteristics (age, sex, race/ethnicity, marital status, income, total wealth, level of education, health insurance, geographic region), childhood abuse, personality factors (openness, conscientiousness, extraversion, agreeableness, neuroticism), and all of the predictor variables, including: health behaviors (physical activity, smoking, overweight/obese, heavy drinking, sleep problems), physical health (heart disease, cancer, stroke, arthritis, hypertension, diabetes, lung disease, chronic pain, hearing, eyesight, self-rated health, physical functioning limitations, cognitive impairment), social factors (live with spouse, frequency of contact with children, frequency of contact with other family, frequency of contact with friends, loneliness, closeness with spouse, number of close children, number of close other family, number of close friends, positive social support from spouse, positive social support from children, positive social support from friends, positive social support from other family, social strain from spouse, social strain from children, social strain from other family, social strain from friends, religious service attendance, volunteering, helping friends/neighbors/relatives, perceived social status, change in perceived social status), psychological well-being factors (life satisfaction, positive affect, optimism, purpose in life, health mastery, financial mastery, mastery), psychological distress (depressive symptoms, hopelessness, negative affect, constraints, anxiety, trait anger, state anger, daily discrimination, major discrimination, cynical hostility, stressful life events, financial strain), and work (in labor force).

^b^All continuous candidate antecedents were standardized (mean = 0; standard deviation = 1).

^c^An exposure-wide analytic approach was used, and a separate model for each exposure was run. Because volunteering was an ordinal outcome, we ran an ordinal logistic regression model to estimate an OR.

^d^These analyses were not included in the main paper as their results are more difficult to interpret (as these analyses represent movement between subcategories of volunteering).

**Supplementary Table 13. Candidate Predictors of Volunteering – Ordinal Volunteering Variable (4 categories): Among People Who Do Not Volunteer at Baseline (Health and Retirement Study [HRS]: N = 7,357)^a,b,c,d^**

| Candidate Predictor | Odds Ratio | 95% CI |
| --- | --- | --- |
| **Health Behaviors** |  |  |
| Frequent physical activity | 1.19 | 0.98, 1.44 |
| Smoking | 0.87 | 0.52, 1.46 |
| Heavy drinking | 1.10 | 0.69, 1.76 |
| Sleep problems | 0.85 | 0.62, 1.17 |
| **Physical Health** |  |  |
| Number of physical conditions | 0.98 | 0.81, 1.19 |
| Diabetes | 0.88 | 0.58, 1.34 |
| Hypertension | 1.16 | 0.84, 1.60 |
| Stroke | 0.64 | 0.35, 1.18 |
| Cancer | 1.17 | 0.66, 2.06 |
| Heart disease | 0.96 | 0.63, 1.48 |
| Lung disease | 0.87 | 0.50, 1.51 |
| Arthritis | 1.20 | 0.85, 1.70 |
| Overweight/obese | 0.89 | 0.67, 1.18 |
| Physical functioning limitations | 0.89 | 0.70, 1.13 |
| Cognitive impairment | 0.79 | 0.60, 1.04 |
| Chronic pain | 1.12 | 0.90, 1.40 |
| Self-rated health | 0.93 | 0.83, 1.05 |
| Hearing | 1.02 | 0.90, 1.16 |
| Eyesight | 1.05 | 0.94, 1.17 |
| **Psychological Well-being** |  |  |
| Positive affect | 1.12 | 1.00, 1.27 |
| Life satisfaction | 1.10 | 0.97, 1.23 |
| Optimism | 1.02 | 0.91, 1.16 |
| Purpose in life | 1.18 | 1.03, 1.35* |
| Mastery | 1.03 | 0.93, 1.15 |
| Health mastery | 0.99 | 0.86, 1.14 |
| Financial mastery | 0.99 | 0.87, 1.12 |
| **Psychological Distress** |  |  |
| Depression | 0.97 | 0.72, 1.31 |
| Depressive symptoms | 0.96 | 0.85, 1.08 |
| Hopelessness | 0.96 | 0.84, 1.09 |
| Negative affect | 1.01 | 0.89, 1.16 |
| Constraints | 0.89 | 0.78, 1.01 |
| Anxiety | 1.00 | 0.89, 1.12 |
| Trait anger | 1.02 | 0.90, 1.16 |
| State anger | 0.96 | 0.87, 1.07 |
| Cynical hostility | 0.95 | 0.86, 1.06 |
| Stressful life events | 1.10 | 1.01, 1.20* |
| Financial strain | 1.02 | 0.89, 1.16 |
| Daily discrimination | 1.02 | 0.91, 1.14 |
| Major discrimination | 1.14 | 1.02, 1.28* |
| **Social Factors** |  |  |
| Living with spouse/partner | 0.79 | 0.56, 1.12 |
| Contact children |  |  |
| <Every few months | Reference | Reference |
| 1-2x/month | 0.98 | 0.52, 1.84 |
| 1-2x/week | 1.50 | 0.88, 2.56 |
| >3x/week | 1.15 | 0.66, 1.99 |
| Contact other family |  |  |
| <Every few months | Reference | Reference |
| 1-2x/month | 0.90 | 0.64, 1.25 |
| 1-2x/week | 0.95 | 0.70, 1.29 |
| >3x/week | 1.12 | 0.84, 1.50 |
| Contact friends |  |  |
| <Every few months | Reference | Reference |
| 1-2x/month | 1.40 | 0.89, 2.19 |
| 1-2x/week | 1.45 | 1.07, 1.98* |
| >3x/week | 1.68 | 1.18, 2.39** |
| Loneliness | 1.04 | 0.92, 1.17 |
| Closeness with spouse | 0.98 | 0.75, 1.29 |
| Number of close children | 1.01 | 0.90, 1.14 |
| Number of close other family | 1.09 | 1.00, 1.18* |
| Number of close friends | 1.04 | 0.93, 1.15 |
| Positive social support from spouse | 1.08 | 0.89, 1.32 |
| Positive social support from children | 1.12 | 1.00, 1.26* |
| Positive social support from other family | 1.08 | 0.97, 1.21 |
| Positive social support from friends | 1.13 | 1.02, 1.26* |
| Social strain from spouse | 0.94 | 0.82, 1.07 |
| Social strain from children | 0.98 | 0.87, 1.11 |
| Social strain from other family | 1.08 | 0.98, 1.20 |
| Social strain from friends | 1.06 | 0.94, 1.20 |
| Religious service attendance |  |  |
| Not at all | Reference | Reference |
| <1x/week | 1.56 | 1.19, 2.05** |
| >1x/week | 2.62 | 1.88, 3.65*** |
| Helping friends/neighbors/relatives |  |  |
| 0 h/year | Reference | Reference |
| 1-49 h/year | 1.35 | 1.06, 1.71* |
| 50-99 h/year | 1.52 | 1.12, 2.06** |
| 100-199 h/year | 1.54 | 1.07, 2.21* |
| >200 h/year | 1.20 | 0.78, 1.84 |
| Social status ladder | 1.02 | 0.89, 1.17 |
| Change in social status ladder |  |  |
| Moved down | Reference | Reference |
| No change | 0.90 | 0.61, 1.31 |
| Moved up | 0.96 | 0.55, 1.68 |
| **Work** |  |  |
| In labor force | 0.95 | 0.74, 1.24 |

**P*<.05 before Bonferroni correction; ** *P*<.01 before Bonferroni correction; ****P*<.05 after Bonferroni correction (the *P* value cutoff for Bonferroni correction is

*P* = .05/61 predictors = *P* <.00081968).

Abbreviations: CI, confidence interval; OR, odds ratio.

^~~a~~^The analytic sample was restricted to those who 1) had participated in the pre-baseline wave (2006 or 2008) and 2) reported no volunteering at baseline. Multiple imputation was performed to impute missing data on the exposures, covariates, and outcome. Candidate antecedents were assessed, one at a time, in wave 2 (2010/2012), and the outcome (volunteering) was assessed in wave 3 (2014/2016). The following covariates were controlled for at wave 1 (2006/2008): sociodemographic characteristics (age, sex, race/ethnicity, marital status, income, total wealth, level of education, health insurance, geographic region), childhood abuse, personality factors (openness, conscientiousness, extraversion, agreeableness, neuroticism), and all of the predictor variables, including: health behaviors (physical activity, smoking, overweight/obese, heavy drinking, sleep problems), physical health (heart disease, cancer, stroke, arthritis, hypertension, diabetes, lung disease, chronic pain, hearing, eyesight, self-rated health, physical functioning limitations, cognitive impairment), social factors (live with spouse, frequency of contact with children, frequency of contact with other family, frequency of contact with friends, loneliness, closeness with spouse, number of close children, number of close other family, number of close friends, positive social support from spouse, positive social support from children, positive social support from friends, positive social support from other family, social strain from spouse, social strain from children, social strain from other family, social strain from friends, religious service attendance, volunteering, helping friends/neighbors/relatives, perceived social status, change in perceived social status), psychological well-being factors (life satisfaction, positive affect, optimism, purpose in life, health mastery, financial mastery, mastery), psychological distress (depressive symptoms, hopelessness, negative affect, constraints, anxiety, trait anger, state anger, daily discrimination, major discrimination, cynical hostility, stressful life events, financial strain), and work (in labor force).

^b^All continuous candidate antecedents were standardized (mean = 0; standard deviation = 1).

^c^An exposure-wide analytic approach was used, and a separate model for each exposure was run. Because volunteering was an ordinal outcome, we ran an ordinal logistic regression model to estimate an OR.

^d^These analyses were not included in the main paper as their results are more difficult to interpret (as these analyses represent movement between subcategories of volunteering).

**Supplementary Table 14. Complete-Case Analyses: Candidate Predictors of Volunteering (Health and Retirement Study [HRS]: N ranged from = 3,030 to 5,463)^a,b,c^**

| Candidate Predictor | Risk Ratio | 95% CI |
| --- | --- | --- |
| **Health Behaviors** |  |  |
| Frequent physical activity | 1.14 | 1.01, 1.28* |
| Smoking | 0.78 | 0.56, 1.09 |
| Heavy drinking | 1.10 | 0.86, 1.41 |
| Sleep problems | 0.96 | 0.84, 1.09 |
| **Physical Health** |  |  |
| Number of physical conditions | 0.96 | 0.86, 1.08 |
| Diabetes | 0.93 | 0.76, 1.14 |
| Hypertension | 0.97 | 0.82, 1.15 |
| Stroke | 0.87 | 0.63, 1.20 |
| Cancer | 1.05 | 0.84, 1.30 |
| Heart disease | 1.01 | 0.83, 1.23 |
| Lung disease | 0.66 | 0.45, 0.98* |
| Arthritis | 1.08 | 0.90, 1.29 |
| Overweight/obese | 0.99 | 0.86, 1.15 |
| Physical functioning limitations | 0.88 | 0.75, 1.03 |
| Cognitive impairment | 0.78 | 0.65, 0.93** |
| Chronic pain | 0.95 | 0.85, 1.06 |
| Self-rated health | 1.06 | 0.99, 1.14 |
| Hearing | 1.00 | 0.94, 1.07 |
| Eyesight | 1.03 | 0.97, 1.09 |
| **Psychological Well-being** |  |  |
| Positive affect | 1.08 | 1.01, 1.16* |
| Life satisfaction | 1.03 | 0.97, 1.10 |
| Optimism | 1.03 | 0.97, 1.10 |
| Purpose in life | 1.05 | 0.98, 1.12 |
| Mastery | 1.03 | 0.97, 1.10 |
| Health mastery | 1.03 | 0.96, 1.10 |
| Financial mastery | 1.02 | 0.96, 1.09 |
| **Psychological Distress** |  |  |
| Depression | 0.89 | 0.72, 1.09 |
| Depressive symptoms | 0.95 | 0.88, 1.02 |
| Hopelessness | 0.94 | 0.88, 1.02 |
| Negative affect | 0.98 | 0.91, 1.05 |
| Constraints | 0.92 | 0.86, 0.99* |
| Anxiety | 0.94 | 0.87, 1.01 |
| Trait anger | 0.99 | 0.94, 1.05 |
| State anger | 0.96 | 0.89, 1.03 |
| Cynical hostility | 0.97 | 0.91, 1.04 |
| Stressful life events | 1.00 | 0.95, 1.06 |
| Financial strain | 1.00 | 0.93, 1.06 |
| Daily discrimination | 0.99 | 0.92, 1.05 |
| Major discrimination | 1.03 | 0.97, 1.10 |
| **Social Factors** |  |  |
| Living with spouse/partner | 0.84 | 0.69, 1.00 |
| Contact children |  |  |
| <Every few months | Reference | Reference |
| 1-2x/month | 1.01 | 0.78, 1.30 |
| 1-2x/week | 1.14 | 0.90, 1.46 |
| >3x/week | 1.07 | 0.84, 1.37 |
| Contact other family |  |  |
| <Every few months | Reference | Reference |
| 1-2x/month | 1.08 | 0.94, 1.24 |
| 1-2x/week | 1.08 | 0.94, 1.25 |
| >3x/week | 1.11 | 0.95, 1.31 |
| Contact friends |  |  |
| <Every few months | Reference | Reference |
| 1-2x/month | 1.33 | 1.08, 1.65** |
| 1-2x/week | 1.40 | 1.14, 1.71** |
| >3x/week | 1.47 | 1.19, 1.81*** |
| Loneliness | 1.01 | 0.95, 1.08 |
| Closeness with spouse | 0.98 | 0.91, 1.05 |
| Number of close children | 1.01 | 0.96, 1.06 |
| Number of close other family | 1.02 | 0.97, 1.06 |
| Number of close friends | 1.04 | 0.99, 1.09 |
| Positive social support from spouse | 1.03 | 0.96, 1.11 |
| Positive social support from children | 1.02 | 0.95, 1.09 |
| Positive social support from other family | 1.00 | 0.94, 1.06 |
| Positive social support from friends | 1.04 | 0.98, 1.11 |
| Social strain from spouse | 0.97 | 0.91, 1.04 |
| Social strain from children | 0.99 | 0.93, 1.05 |
| Social strain from other family | 1.01 | 0.95, 1.07 |
| Social strain from friends | 1.05 | 0.99, 1.11 |
| Religious service attendance |  |  |
| Not at all | Reference | Reference |
| <1x/week | 1.63 | 1.33, 1.99*** |
| >1x/week | 2.27 | 1.82, 2.83*** |
| Helping friends/neighbors/relatives |  |  |
| 0 h/year | Reference | Reference |
| 1-49 h/year | 1.17 | 1.04, 1.32* |
| 50-99 h/year | 1.29 | 1.12, 1.48*** |
| 100-199 h/year | 1.26 | 1.07, 1.48** |
| >200 h/year | 1.20 | 1.00, 1.44 |
| Social status ladder | 1.05 | 0.98, 1.12 |
| Change in social status ladder |  |  |
| Moved down | Reference | Reference |
| No change | 0.96 | 0.83, 1.12 |
| Moved up | 0.99 | 0.80, 1.22 |
| **Work** |  |  |
| In labor force | 1.03 | 0.90, 1.17 |

**P*<.05 before Bonferroni correction; ** *P*<.01 before Bonferroni correction; ****P*<.05 after Bonferroni correction (the *P* value cutoff for Bonferroni correction is *P* = .05/61 predictors = *P* <.00081968).

Abbreviations: CI, confidence interval; RR, risk ratio.

^~~a~~^The analytic sample was restricted to those who had participated in the pre-baseline wave (2006 or 2008). Candidate antecedents were assessed, one at a time, in wave 2 (2010/2012), and the outcome (volunteering) was assessed in wave 3 (2014/2016). The following covariates were controlled for at wave 1 (2006/2008): sociodemographic characteristics (age, sex, race/ethnicity, marital status, income, total wealth, level of education, health insurance, geographic region), childhood abuse, personality factors (openness, conscientiousness, extraversion, agreeableness, neuroticism), and all of the predictor variables, including: health behaviors (physical activity, smoking, overweight/obese, heavy drinking, sleep problems), physical health (heart disease, cancer, stroke, arthritis, hypertension, diabetes, lung disease, chronic pain, hearing, eyesight, self-rated health, physical functioning limitations, cognitive impairment), social factors (live with spouse, frequency of contact with children, frequency of contact with other family, frequency of contact with friends, loneliness, closeness with spouse, number of close children, number of close other family, number of close friends, positive social support from spouse, positive social support from children, positive social support from friends, positive social support from other family, social strain from spouse, social strain from children, social strain from other family, social strain from friends, religious service attendance, volunteering, helping friends/neighbors/relatives, perceived social status, change in perceived social status), psychological well-being factors (life satisfaction, positive affect, optimism, purpose in life, health mastery, financial mastery, mastery), psychological distress (depressive symptoms, hopelessness, negative affect, constraints, anxiety, trait anger, state anger, daily discrimination, major discrimination, cynical hostility, stressful life events, financial strain), and work (in labor force).

^b^All continuous candidate antecedents were standardized (mean = 0; standard deviation = 1).

^c^An exposure-wide analytic approach was used, and a separate model for each exposure was run. Because volunteering was a binary outcome with a prevalence of ≥10%, we ran a generalized linear model with a log link and Poisson distribution to estimate a RR.
